# Trends and determinants of healthcare-induced poverty in China 2013–2019

**DOI:** 10.1093/heapol/czaf026

**Published:** 2025-06-12

**Authors:** Linwei Li, Bingqing Guo, Chaojie Liu, Qiang Yao

**Affiliations:** School of Political Science and Public Administration, Wuhan University, No. 299 Bayi Road, Wuchang District, Wuhan 430072, China; School of Public Health, Imperial College London, 90 Woodlane, London W12 0BZ, United Kingdom; School of Public Health, Li Ka Shing Faculty of Medicine, The University of Hong Kong, No. 7 Sasson Road, Pok Fu Lam, Hong Kong SAR 999077, China; School of Psychology and Public Health, La Trobe University, 1 Kingsbury Dr, Melbourne VIC 3086, Australia; School of Political Science and Public Administration, Wuhan University, No. 299 Bayi Road, Wuchang District, Wuhan 430072, China

**Keywords:** healthcare-induced poverty, catastrophic health expenditure, impoverishment health expenditure, China

## Abstract

Healthcare costs are a major driver of poverty, accounting for 44.1% of poverty cases in China. By 2015, nearly 20 million people fell into or returned to poverty due to health issues. In response, the Chinese government launched the national health poverty alleviation project in 2016. This study aims to evaluate the distribution and trends of healthcare-induced poverty from 2013 to 2019. Using data from the China Household Finance Survey (CHFS), we estimated the incidence of household catastrophic health expenditure (CHE) and impoverishing health expenditure (IHE) and analyzed their determinants through multi-level logistic regression models. Subgroup analyses were conducted based on rural/urban location, geographic region, and province. In 2013, 31.83% of households experienced CHE, while 9.56% faced IHE. CHE incidence declined significantly after 2016 [adjusted odds ratio (AOR)  = 0.493–0.766, *P* < 0.001]. IHE incidence initially increased in 2015 (AOR = 1.580, *P* < 0.001) before declining from 2017 onward (AOR = 0.465–0.607, *P* < 0.001). The most significant reduction (9.99%–10.95%) occurred among the highest income quartile. CHE and IHE shared similar determinants. Higher odds of CHE and IHE were associated with older age of the household head (AOR = 1.225–2.175, *P* < 0.001), rural residency (AOR = 1.093–1.199, *P* < 0.05), the presence of an elderly household member (AOR = 1.237–1.336, *P* < 0.001), and having more household members in poor self-rated health (AOR = 2.455–4.137, *P* < 0.001). Conversely, lower odds of CHE and IHE were associated with higher educational attainment (AOR = 0.681–0.879, *P* < 0.001) and employment (AOR = 0.610–0.708, *P* < 0.001) of the household head, higher household income per capita (AOR = 0.017–0.860, *P* < 0.001), and larger household size (AOR = 0.335–0.684, *P* < 0.001). Households in urban areas and the eastern developed region had lower incidences of CHE and IHE compared to others. In conclusion, China has seen a significant decline in CHE and IHE, particularly after implementing the national poverty alleviation project. However, regional, urban–rural, and income-related disparities persist, underscoring the need for equity-focused interventions.

Key messages

- Diseases are a significant driver of poverty in China, prompting the government to implement health poverty alleviation projects from 2016 to 2020. However, limited research has examined changes in healthcare-induced poverty during the policy period.
- Following the implementation of health poverty alleviation projects, the odds of catastrophic health expenditure and impoverishing health expenditure decreased compared to 2013 levels.
- While financial support and strengthened health management contribute to alleviating healthcare-induced poverty, optimizing public funding effectiveness is crucial, as lowering financial barriers may increase healthcare utilization and expenditure.
- The incidence of healthcare-induced poverty remains higher in rural areas and less developed regions of central and western China, underscoring the need for equity-focused interventions in health poverty alleviation efforts.

## Introduction

Poverty has always been a global concern. The United Nations (UN) established 17 sustainable development goals (SDGs) to be achieved by 2030, with poverty eradication as the primary objective (United Nations 2015). Health and healthcare services have an impact on poverty through multiple pathways. Poor health can diminish an individual’s ability to earn income, thereby increasing the risk of poverty (Li et al. 2023). High out-of-pocket health expenditure can lead to financial hardship when it exceeds a certain threshold of a household’s consumption or income (Sirag and Mohamed Nor 2021). Additionally, poor health itself is regarded as a form of poverty resulting in capability deprivation (Deng et al. 2015). The UN emphasized the importance of health services in achieving the goal of ending poverty and further reaffirmed that universal health coverage would be beneficial to long-term economic development (World Health Organization 2023). Empirical evidence shows that the transfer of a productive asset, food, or cash had potentially positive effects on the health of extremely poor people in six developing countries (Banerjee et al. 2015).

Preventing healthcare-induced poverty is a critical global policy objective aligned with the SDGs. Efforts to alleviate poverty generally focus on three key approaches: improving healthcare utilization, reducing out-of-pocket health expenditure, and providing financial support to impoverished populations. Enhancing healthcare utilization helps improve health outcomes among disadvantaged groups, addressing healthcare-induced poverty at its root. For example, India’s Ayushman Bharat program established 150 000 health and wellness centers in impoverished areas to ensure access to essential healthcare services (Bhargava and Paul 2022). Mexico established the *Seguro Popular*, a health insurance program with no premium requirements for low-income families (Parker et al. 2018). Indonesia (Agustina et al. 2019) and Thailand (Sumriddetchkajorn et al. 2019) have developed comprehensive programs to mitigate healthcare-induced poverty, with a particular focus on disadvantaged populations.

Healthcare-induced poverty (referred to as health poverty) has been a severe problem in China. In total, 44.1% of China’s poverty was observed to be caused by diseases, and nearly 20 million people fell into or returned to poverty due to health problems, of whom 3.3 million suffered from serious illnesses and 4 million suffered from long-term chronic conditions (State Council 2018a). In 2016, China’s National Health and Family Planning Commission launched the national health poverty alleviation project, requiring all provinces to implement it (State Council 2016c). This initiative aimed to ensure that impoverished populations had access to essential healthcare services while preventing other households from falling into poverty due to illness. The health poverty alleviation project became a key component of the Healthy China Initiative and the broader poverty eradication campaign. Targeting disadvantaged populations and regions, its main actions included strengthening financial protection related to healthcare, controlling health expenditure for severe illnesses, providing treatment for chronic diseases, and enhancing healthcare service delivery (State Council, 2016c).

**Figure 1.**
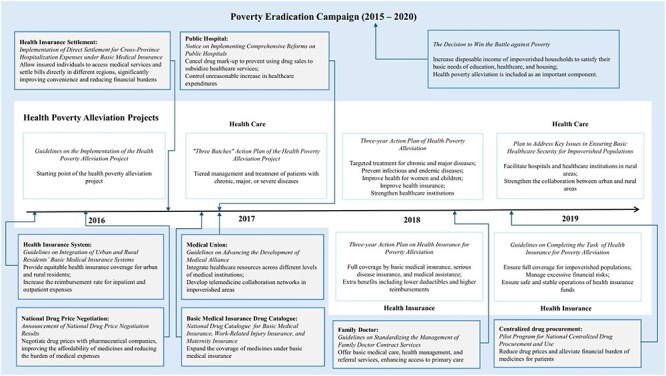
Major policies relevant to health poverty alleviation.

*Note: The light blue (white) boxes represent key policies trigged by the health poverty alleviation project, while the dark blue (grey) boxes represent other relevant health policies implemented.*

Multiple policies were developed under the health poverty alleviation project and the broader poverty eradication campaign (Fig. 1). The health poverty alleviation project aims to reduce healthcare expenditure by reinforcing financial protection, improving disease management and treatment (National Health Commission 2017a), and strengthening healthcare infrastructure in impoverished regions (State Council 2019c). Universal social health insurance coverage, along with additional medical assistance for low-income populations—such as lower deductibles and higher reimbursement rates—plays a crucial role in this initiative (State Council 2018c, State 2019a). Beyond health insurance reforms (MOHRSS 2016, State Council 2016b), several complementary reforms were introduced, including those targeting hospitals (National Health Commission 2017b), healthcare alliance (State Council 2017), family doctors (State Council 2018b), drug price negotiation (National Health Commission 2016), and centralized drug procurement (State Council 2019b). These measures collectively aim to alleviate the financial burden of healthcare. Meanwhile, the broader poverty eradication campaign expanded its efforts by providing comprehensive economic support to impoverished households, increasing their disposable income to meet essential needs (State Council 2015). These initiatives have been implemented across various sectors, including agriculture, tourism, vocational education, and employment services (State Council 2016a). Detailed information on the mentioned policies can be found in Appendix 1 (see online supplementary material).

Extensive studies have examined the incidence of catastrophic health expenditure (CHE) and impoverishing health expenditure (IHE) in China (Ma et al. 2019, Zhao et al. 2020, Jia et al. 2023), the two key indicators of health poverty alleviation efforts. However, there is a notable gap in the literature regarding changes in CHE and IHE during the critical period of China’s poverty eradication campaign (2015–2020). Existing studies either fall outside this pivotal timeframe (Ma et al. 2019, Zhao et al. 2020) or provide only a cross-sectional view without tracking trends over time (Jia et al. 2023). Additionally, few studies compare regional variations (Chen and J 2019, Lu et al. 2021) or population differences (Ma et al. 2020, Qin et al. 2021), limiting their relevance for policy action.

Our research addresses gaps in the literature by analyzing trends in healthcare-induced poverty, covering the period of the health poverty alleviation project, and examining regional variations and population differences. Unlike previous studies, we estimate both CHE and IHE indicators to capture the multidimensional nature of health poverty. By linking findings to household- and provincial-level factors over time, this study provides a comprehensive assessment of health poverty, offering insights into targeted interventions and sustainable poverty reduction strategies.

## Methods

### Data sources

The household-level data were obtained from the China Household Finance Survey (CHFS), a nationally conducted project by the Survey and Research Centre for China Household Finance. It employed a multi-stage stratified sampling strategy to recruit study participants, covering 29 out of the 34 provinces/regions in China (Southwestern University Of Finance and Economics 2022). A total of 140 084 household responses were valid for data analysis: 28 141 from 2013, 37 289 from 2015, 40 011 from 2017, and 34 643 from 2019.

The provincial-level data were extracted from the China Health Statistical Yearbooks (2013–2019) (National Health Commission 2023) and the China Statistical Yearbooks (2013–2019) (National Bureau Of Statistics 2023). They were matched to each household according to the year and location of the surveyed household.

### Variables

#### Dependent variables

CHE proposed by the World Health Organization (WHO) defines out-of-pocket health expenditure as catastrophic when it equals or exceeds 40% of a household’s non-subsistence income (i.e. income available after basic needs have been met) (Xu et al. 2005). The UN SDGs define CHE as out-of-pocket health expenditure greater than 10% or 25% of total household expenditure or income (United Nations 2023).

IHE measures household consumption per capita against the poverty line: it pushes household consumption per capita from above the poverty line to below it after subtracting per capita out-of-pocket health expenditure (Grépin et al. 2020). The choice of the poverty line is often contextualized.

This study utilized both CHE and IHE to measure health poverty. We applied the CHE definitions from the WHO and the UN SDGs. IHE was estimated using both the international poverty line and the China poverty line (Table 1).

**Table 1.** Definitions of CHE and IHE

	Definition	Threshold
CHE1	Health expenditure ≥40% of a household’s income minus food expenditure	
CHE2	Health expenditure ≥25% of total household income	
IHE1	Healthcare-induced poverty against the poverty line set by the Chinese government	2013: Ұ2 736 2015: Ұ2 855 2017: Ұ2 952 2019: Ұ3 218
IHE2	Healthcare-induced poverty against the poverty line set by the World Bank Conversion using the purchasing power parity method	2013: Ұ1 770 2015: Ұ2 684 2017: Ұ2 899 2019: Ұ2 920

Supplementary Table S1, see online supplementary material, provides detailed information on the measurement and data sources for household consumption, income, and health expenditure.

#### Independent and control variables

‘Year’ was introduced in the statistical modelling as an independent variable to measure trends. China initiated a poverty eradication campaign in 2015 and subsequently the health poverty alleviation project in 2016. The data (2013–2019) used in this study covered the time periods before and after the poverty eradication campaign and the health poverty alleviation project.

Both household- and provincial-level factors were considered in the statistical modeling. The selection of household-level variables was guided by Andersen’s Behavioral Model of Health Services Use, a widely used theoretical framework for explaining the determinants of CHE (Brinda et al. 2014). This model categorizes the determinants of health service utilization into predisposing, enabling, and need factors. Following recent systematic reviews (Borde et al. 2022, Eze et al. 2022) and aligning with Andersen’s model, this study measured the following predictors of health poverty. (i) Predisposing factors—gender and age of the household head, and household size. (ii) Enabling factors—educational attainment and employment status of the household head, annual income per capita, coverage by different types of health insurance, coverage by pension insurance for the elderly, and the household’s regional and urban/rural location. (III) Need factors—the number of household members with self-rated poor health and the presence of household members with high healthcare needs, such as children or the elderly (Wang et al. 2024) (Table 2).

**Table 2.** Independent and control variables included in the regression models

Variable	Categorization
Year	2013, 2015, 2017, 2019
**Household level**	
**Predisposing factor**	
Gender of household head	Female, male
Age (years) of household head	<45, 45–54, 55–65, >65
Household size	<3, 3, 4, >4
**Enabling factor**	
Educational attainment of household head	Up to primary school, junior middle school, senior middle school, tertiary education
Employment of household head	Unemployed, employed
Annual household income per capita	Quartiles within each year: lowest, lower, higher, highest
Basic medical insurance	(i) None: no household member covered by basic medical insurance; (ii) partial coverage: some household members, but not all, covered by basic medical insurance; (iii) full coverage by the UEBMI: all household members covered by the basic medical insurance for urban employees; (iv) full coverage by the URRBMI: all household members covered by the basic medical insurance for urban and rural residents; (v) full mixed coverage: all household members covered by basic medical insurance, but with different types.
Commercial medical insurance	No, yes
Households with age pension for employees	Household members aged ≥60 years covered by age pension for employees (no, yes)
Residency	Urban, rural
**Need factor**	
Household with elderly	Household with members aged ≥60 years (no, yes)
Household with children	Household with members aged ≤5 years (no, yes)
Number of members in poor health	Household members with self-rated poor or very poor health (0, 1, >1)
**Provincial-level variable**	
Health expenditure per capita	Continuous variable, per capita total health expenditure by province
Percentage of total government expenditure on health	Continuous variable, proportion of government health expenditure in total health expenditure by province
Number of beds in medical institutions per 1000 population	Continuous variable, number of health institution beds per 1000 population by province
Annual hospitalization rate	Continuous variable, annual hospitalization rate by province
Average number of doctor visits by residents	Continuous variable, annual average number of doctor visits per resident by province

The provincial-level variables included average health expenditure per capita, the governmental share of total health expenditure, hospital beds per 1000 population, the annual hospitalization rate, and the average number of medical visits per person per year. These variables capture both the demand and supply aspects of healthcare services (Eze et al. 2022, Topmiller et al. 2023).

### Statistical analysis

The incidence of CHE and IHE was calculated for the entire sample and for subgroups to assess disparities in their distribution and identify which groups experienced the most significant changes during the study period (2013–2019).

Multilevel logistic regression models were established to determine the associations of household- and provincial-level factors with the incidence of CHE and IHE. The household samples in this study are distributed across 29 provincial administrative units, with each provincial unit comprising a sample of households ranging from 687 to 1528 and forming a two-level data structure.

We assessed the variance inflation factor for all predictors and confirmed the absence of multicollinearity (all variance inflation factor < 5) (O’Brien 2007).

An empty model was established first to determine the provincial cluster effects using likelihood ratio (LR) tests. The *P*-value of the LR test for the empty model for both CHE and IHE was <0.001, indicating the necessity of an unconstrained multilevel model (Peugh 2010).

We established two-level logistic regression models for CHE and IHE, respectively. The Akaike Information Criterion for the two-level CHE and IHE models was consistently lower than that of their corresponding empty model, indicating the appropriateness of the two-level models. In this study, we did not examine the correlations between household and provincial variables.

Stata 16.0 IC was used in the statistical analyses.

## Results

### Characteristics of respondents

More than three-quarters of household heads were male; around half were >55 years old; ∼15% had a university degree; and >60% were employed. Around 40% of households had three or four members; half had a member >60 years old; <15% had a child (Table 3).

**Table 3.** Characteristics of study participants

Variable	2013		2015		2017		2019	
	*n*	%	*n*	%	*n*	%	*n*	%
**Household level**	28 141		37 289		40 011		34 643	
**Predisposing factor (household head)**								
Gender								
Female	6 843	24.32	9 133	24.49	8 272	20.67	8 540	24.65
Male	21 297	75.68	28 156	75.51	31 738	79.33	26 103	75.35
Age (years)								
<45	9 485	33.71	10 440	28.00	9 187	22.96	6 767	19.53
45–54	7 022	24.95	9 826	26.35	10 495	26.23	8 708	25.14
55–65	6 788	24.12	9 355	25.09	10 441	26.10	9 614	27.75
>65	4 846	17.22	7 668	20.56	9 888	24.71	9 554	27.58
**Predisposing factor (household)**								
Household size								
<3	8 168	29.03	10 663	28.60	16 169	40.41	15 531	44.83
3	8 113	28.83	10 110	27.11	10 051	25.12	7 806	22.53
4	5 257	18.68	6 844	18.35	6 112	15.28	5 086	14.68
>4	6 603	23.46	9 672	25.94	7 679	19.19	6 220	17.95
**Enabling factor (household head)**								
Educational attainment								
Up to primary school	8 622	30.65	11 829	31.77	12 575	31.47	11 177	32.30
Junior middle school	9 233	32.83	12 180	32.71	13 237	33.13	11 898	34.38
Senior middle school	5 615	19.96	7 281	19.55	7 793	19.50	6 607	19.09
Tertiary education	4 657	16.56	5 947	15.97	6 353	15.90	4 922	14.22
Employment								
Unemployed	9 184	32.65	13 071	35.17	15 345	38.37	752	20.18
Employed	1 8945	67.35	24 098	64.83	24 651	61.63	2 975	79.82
**Enabling factor (household)**								
Annual household income per capita								
Lowest quintile	7 035	25.00	9 338	25.04	10 004	25.00	8 661	25.00
Lower	7 058	25.08	9 314	24.98	10 003	25.00	8 660	25.00
Higher	7 013	24.92	9 338	25.04	10 002	25.00	8 662	25.00
Highest quintile	7 035	25.00	9 299	24.94	10 002	25.00	8 660	25.00
Basic medical insurance								
None	2 429	8.64	2 455	6.58	1 631	4.08	1 292	3.73
Partial coverage	19 531	69.44	11 684	31.33	6 627	16.56	4 625	13.35
Full coverage by UEBMI	2 064	7.34	3 817	10.24	5 171	12.92	4 388	12.67
Full coverage by URRBMI	3 006	10.69	14 180	38.03	19 086	47.70	17 904	51.68
Full coverage by mixed	1 098	3.90	5 153	13.82	7 496	18.73	6 434	18.57
Commercial medical insurance								
No	26 557	94.37	35 346	94.79	37 929	94.80	32 366	93.43
Yes	1 584	5.63	1 943	5.21	2 082	5.20	2 277	6.57
Age pension for employees								
No	24 092	85.61	30 614	82.10	31 581	78.93	27 925	80.61
Yes	4 049	14.39	6 675	17.90	8 430	21.07	6 718	19.39
Residency								
Urban	19 209	68.26	25 635	68.75	27 279	68.18	22 307	64.39
Rural	8 932	31.74	11 654	31.25	12 732	31.82	12 336	35.61
**Need**								
Household with elderly								
Yes	12 920	45.91	19 225	51.56	21 958	54.88	19 603	56.59
No	15 221	54.09	18 064	48.44	18 053	45.12	15 040	43.41
Household with children								
Yes	4 075	14.48	4 981	13.36	4 588	11.47	3 649	10.53
No	24 066	85.52	32 308	86.64	35 423	88.53	30 994	89.47
Number of members in poor health								
0	9 947	35.35	25 973	69.65	26 692	66.71	22 664	65.42
1	8 560	30.42	7 763	20.82	8 641	21.60	7 468	21.56
>1	9 634	34.23	3 553	9.53	4 678	11.69	4 511	13.02
**Provincial level**	*n*	Mean ± SD	*n*	Mean ± SD	*n*	Mean ± SD	*n*	Mean ± SD
Health expenditure per capita	29	2 346.46 ± 1 027.77	29	2 912.00 ± 1 147.09	29	3 795.71 ± 1 533.98	29	4 475.44 ± 1 767.79
Percentage of total government expenditure on health (%)	29	30.31 ±6.30	29	29.40 ±6.40	29	29.14 ±6.12	29	28.40 ±5.97
Number of hospital beds per 1000 population	29	4.28 ±0.48	29	4.83 ±0.59	29	5.37 ±0.68	29	6.00 ±0.80
Annal hospitalization rate (%)	29	12.80 ±2.05	29	14.43 ±2.29	29	15.93 ±2.52	29	17.74 ±3.07
Annal average number of medical visits	29	5.32 ±1.75	29	5.80 ±1.92	29	6.03 ±2.11	29	5.97 ±2.10

*Note:* UEBMI,urban employee basic medical insurance; URRBMI, Urban and rural resident basic medical insurance; SD, standard deviation.

The majority (≥65%) of households were located in urban areas. Coverage of basic medical insurance increased over time, with > 96% of households having at least one insured member by 2019. However, <7% of households purchased commercial health insurance. Less than 20% of households with elderly members received an age pension for employees. The average annual household income per capita reached 27 649 Yuan. Across most years, <35% of household members self-rated their health as poor, except in 2013, when this figure was 64% (Table 3).

At the provincial level, health expenditure per capita almost doubled from 2013 to 2019. The proportion of government expenditure in total health expenditure remained stable at ∼30%. Hospital beds per 1000 population increased slightly by 1.72%. The annual hospitalization rate rose from 12.8% to 17.7% over the study period, while the annual number of medical visits per resident showed a slight increase, rising from 5.32 to 5.97 (Table 3).

### Trend of CHE and IHE

**Figure 2.**
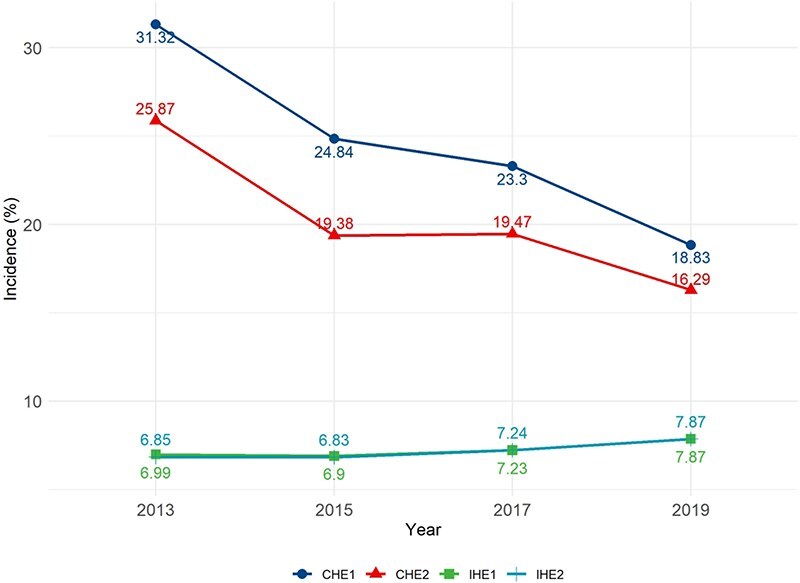
Incidence of CHE and IHE over the period from 2013 to 2019.

*Note: CHE1-CHE using the WHO threshold; CHE2 – CHE using the UN SDGs threshold; IHE1 – IHE against the China poverty line; IHE2 – IHE against the World Bank poverty line*

From 2013 to 2019, the incidence of CHE decreased despite an increase in healthcare service volumes: it decreased from 31.32% in 2013 to 18.83% in 2019 as measured by the WHO threshold and from 25.87% in 2013 to 16.29% in 2019 as measured by the UN SDGs threshold (Fig. 2).

From 2013 to 2019, the incidence of IHE increased slightly: it increased from 6.99% in 2013 to 7.87% in 2019 against the China poverty line and from 6.85% in 2013 to 7.87% in 2019 against the international poverty line (Fig. 2).

**Figure 3.**
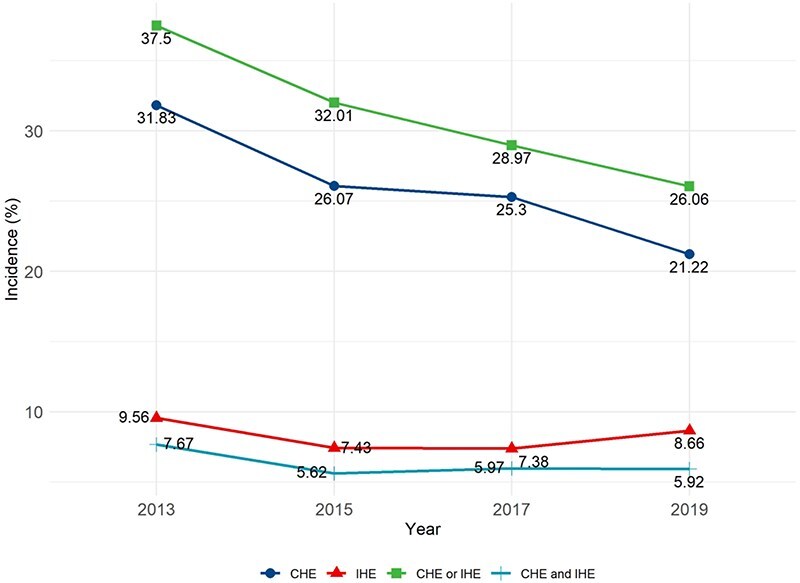
Incidence of CHE and/or IHE over the period from 2013 to 2019.

*Note: CHE – CHE1 (CHE using the WHO threshold) or CHE2 (CHE using the UN SDGs threshold); IHE – IHE1 (IHE against the China poverty line) or IHE2 (IHE against the World Bank poverty line)*

If we include both households with CHE or IHE, the declining trend over time persisted: 37.50% of households incurred either CHE or IHE in 2013, compared with 26.06% in 2019 (Fig. 3).

Inequalities existed in the decline of health poverty, with the most significant reduction (9.99%–10.95%) observed among households in the highest income quartile. In contrast, households with more than one member reporting poor self-rated health experienced an increase in health poverty (0.32%–6.90%) (Table S2, see online supplementary material).

**Figure 4.**
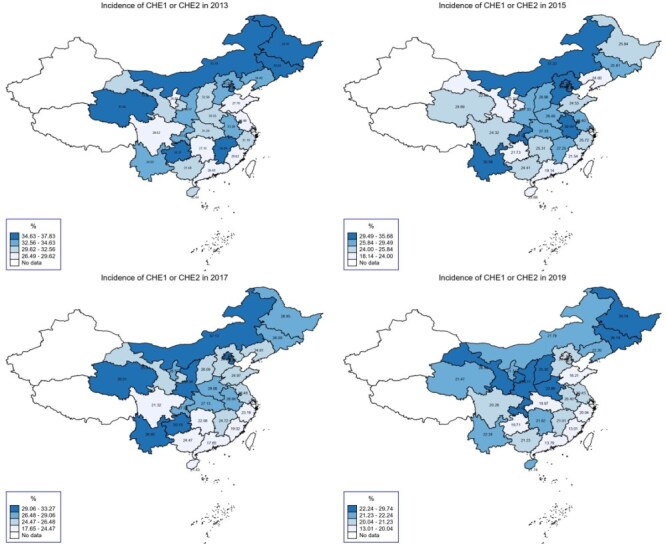
Incidence of CHE by province from 2013 to 2019.

**Figure 5.**
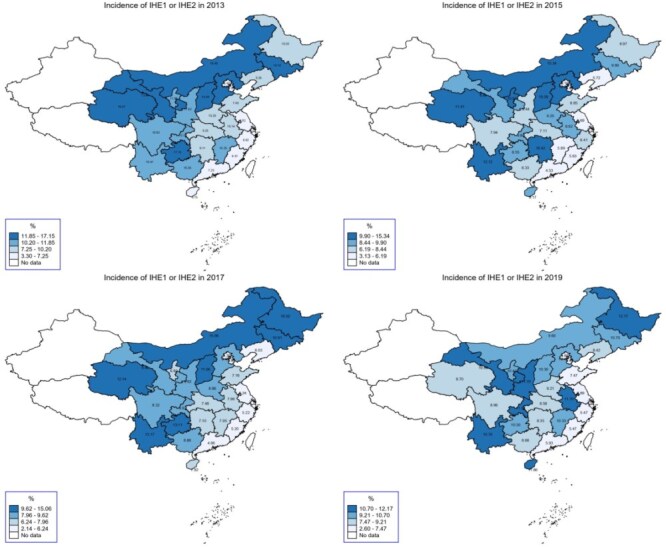
Incidence of IHE by provinces from 2013 to 2019.

The eastern developed region had a lower incidence of CHE and IHE compared to the central and western regions (Figs 4 and 5). Within the eastern developed region, Fujian and Shanghai recorded the lowest incidence of CHE (13.01%) and IHE (2.60%) in 2019, whereas Heilongjiang, from the central developing region, had the highest incidence of CHE (29.74%) and IHE (12.17%) in the same year.

**Figure 6.**
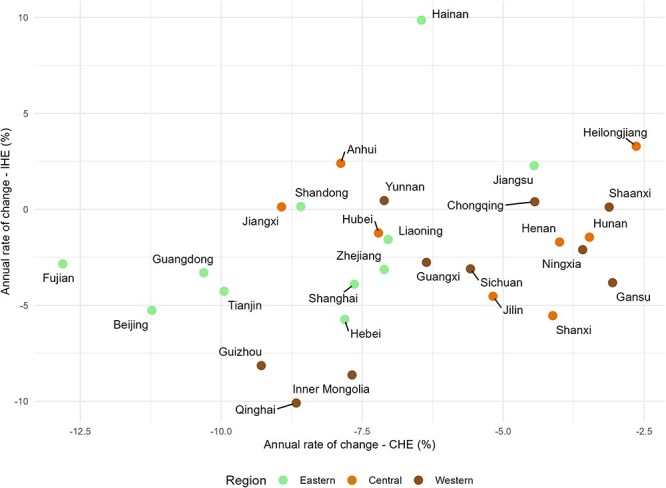
Annual rates of changes in incidence of CHE and IHE by province from 2013 to 2019.

Households in urban areas (6.81%) and the eastern developed region (8.31%) experienced the greatest reductions in CHE, while those in rural areas (2.95%) and the western underdeveloped region (6.81%) saw the most significant decreases in IHE (Table S3, see online supplementary material). Among provinces, Heilongjiang (central developing region) exhibited the lowest annual change in CHE (2.64%), whereas Fujian (eastern developed region) showed the highest (12.81%). Hainan, also in the eastern developed region, experienced a concerning rise in IHE at a rate of 9.86%, whereas Qinghai (western underdeveloped region) recorded the largest decline (10.09%) in IHE (Fig. 6).

Lower annual change rates were associated with higher incidences of CHE and IHE (Figs S1 and S2, respectively, see online supplementary material) in 2019.

### Factors associated with CHE

The regression models indicated that there was no decline in the incidence of CHE measured by the WHO threshold between 2013 and 2015 (*P* > 0.05) before the launch of the health poverty alleviation project. However, a significant decline has been observed since the project’s initiation in 2016. The odds of CHE measured by the WHO threshold decreased by 30.1% [adjusted odds ratio (AOR) = 0.699, *P* < 0.001] in 2017 and 56.3% (AOR = 0.437, *P* < 0.001) in 2019, respectively, compared with the CHE level in 2013. Similarly, the odds of CHE measured by the UN SDGs threshold decreased by 22.5% (AOR = 0.775, *P* < 0.01) in 2017 and 53.2% (AOR = 0.468, *P* < 0.001) in 2019, respectively, compared with the CHE level in 2013 (Table 4).

**Table 4.** Factors associated with CHE: results of two-level logistic regression models

	CHE1 using WHO threshold		CHE2 using SDGs threshold		CHE1 or CHE2	
	Model 1		Model 2		Model 3	
Variable (reference)	AOR	*P*	AOR	*P*	AOR	*P*
**Year (2013)**						
2015	0.968	0.395	0.961	0.399	1.014	0.719
2017	0.699	<0.001	0.775	0.004	0.766	<0.001
2019	0.437	<0.001	0.468	<0.001	0.493	<0.001
**Predisposing factor (household head)**						
Gender (female)						
Male	1.019	0.327	1.017	0.401	1.017	0.367
Age (<45 years)						
45–54	1.320	<0.001	1.304	<0.001	1.304	<0.001
55–65	1.482	<0.001	1.427	<0.001	1.450	<0.001
>65	2.222	<0.001	2.025	<0.001	2.175	<0.001
**Predisposing factor (household)**						
Household size (<3)						
3	0.673	<0.001	0.700	<0.001	0.684	<0.001
4	0.609	<0.001	0.632	<0.001	0.624	<0.001
>4	0.556	<0.001	0.601	<0.001	0.577	<0.001
**Enabling factors (household head)**						
Educational attainment (up to primary school)						
Junior middle school	0.883	<0.001	0.876	<0.001	0.879	<0.001
Senior middle school	0.826	<0.001	0.844	<0.001	0.818	<0.001
Tertiary education	0.675	<0.001	0.694	<0.001	0.681	<0.001
Employment (unemployment)						
Employed	0.690	<0.001	0.700	<0.001	0.708	<0.001
**Enabling factor (household)**						
Annual household income per capita (lowest quintile)						
Lower	0.954	0.025	0.981	0.384	0.946	0.007
Higher	0.863	<0.001	0.915	<0.001	0.860	<0.001
Highest quintile	0.819	<0.001	0.971	0.321	0.834	<0.001
Basic medical insurance (full coverage by UEBMI)						
None	0.897	0.010	0.918	0.062	0.909	0.023
Partial coverage	0.913	0.007	0.992	0.826	0.919	0.011
Full coverage by URRBMI	0.921	0.010	1.005	0.896	0.944	0.072
Full coverage by mixed	0.906	0.003	0.983	0.629	0.924	0.018
commercial medical insurance (No)						
Yes	0.797	<0.001	0.800	<0.001	0.797	<0.001
Age pension for employees (no)						
Yes	1.267	<0.001	1.149	<0.001	1.259	<0.001
Residency (urban)						
Rural	1.154	<0.001	1.286	<0.001	1.199	<0.001
**Need factor**						
Household with elderly (no)						
Yes	1.325	<0.001	1.410	<0.001	1.336	<0.001
Household with children (no)						
Yes	1.210	<0.001	1.202	<0.001	1.210	<0.001
Number of members in poor self-rated health (0)						
1	2.415	<0.001	2.663	<0.001	2.455	<0.001
>1	3.757	<0.001	4.189	<0.001	3.838	<0.001
**Provincial level variable**						
Health expenditure per capita	1.348	0.032	1.232	0.280	1.346	0.034
Percentage of total government expenditure on health	0.997	0.535	0.990	0.128	0.996	0.426
Number of hospital beds per 1000 population	0.901	0.036	0.902	0.083	0.932	0.149
Annal hospitalization rate	1.015	0.270	1.038	0.056	1.012	0.396
Average number of medical visits per resident	0.985	0.519	0.949	0.058	0.975	0.264
Model statistics						
LR test (*P*)	0.000		0.000		0.000	
AIC	106 040.657		95 621.818		108 881.773	

*Note:* AOR, adjusted odds ratio; UEBMI, urban employee basic medical insurance; URRBMI, Urban and rural resident basic medical insurance; LR test, likelihood ratio test; AIC, Akaike information criterion.

Higher educational attainment (AOR = 0.675–0.883, *P* < 0.001) and employment of household head (AOR = 0.690–0.700, *P* < 0.001), larger household size (AOR = 0.556–0.700, *P* < 0.01), and higher household income (AOR = 0.819–0.915, *P* < 0.01) were protective factors against CHE. While commercial insurance was associated with lower odds (AOR = 0.797–0.800, *P* < 0.001) of CHE, households covered entirely by the Urban Employee Basic Medical Insurance (UEBMI), the most prestigious social health insurance program (with higher funding pools and entitlements), and households with elderly members covered by pension insurance for employees (the most generous pension fund) had higher odds of CHE than others (Table 4).

Older age of household head (AOR = 1.304–2.222, *P* < 0.001), rural location (AOR = 1.154–1.286, *P* < 0.001), household with elderly (AOR = 1.325–1.410, *P* < 0.001) or children (AOR = 1.202–1.210, *P* < 0.001), and more members with self-rated poor health (AOR = 2.415–4.189, *P* < 0.01) were risk factors for CHE (Table 4).

No significant associations between CHE and the provincial-level factors were observed (Table 4).

The regression models for urban and rural areas (Table S4, see online supplementary material), as well as for different regions (Table S5, see online supplementary material), identified similar predictors of CHE, reinforcing the findings from the national sample.

### Factors associated with IHE

The regression models revealed that in 2015 (prior to the launch of the health poverty alleviation project), the incidence of IHE increased by 79.5% (AOR = 1.795, *P* < 0.001) against the China poverty line and by 141.3% (AOR = 2.413, *P* < 0.01) against the World Bank poverty line, respectively, compared to the year 2013. The odds of IHE declined in 2017 by 38.8% (AOR = 0.612, *P* < 0.001) and in 2019 by 28.9% (AOR = 0.711, *P* < 0.05) against the China poverty line (Table 5).

**Table 5.** Factors associated with IHE: results of two-level logistic regression models

	IHE1 using China poverty line		IHE2 using World Bank poverty line		IHE1 or IHE2	
	Model 1		Model 2		Model 3	
Variable (reference)	AOR	*P*	AOR	*P*	AOR	*P*
**Year (2013)**						
2015	1.795	<0.001	2.413	<0.001	1.580	<0.001
2017	0.612	<0.001	0.910	0.198	0.465	<0.001
2019	0.711	0.019	0.945	0.682	0.607	<0.001
**Predisposing factor (household head)**						
Gender (female)						
Male	0.949	0.159	0.961	0.281	0.961	0.280
Age (<45 years)						
45–54	1.045	0.376	1.032	0.524	1.045	0.357
55–65	0.999	0.988	0.995	0.923	1.009	0.861
>65	1.247	<0.001	1.227	0.001	1.255	<0.001
**Predisposing factor (household)**						
Household size (<3)						
3	0.537	<0.001	0.533	<0.001	0.530	<0.001
4	0.417	<0.001	0.419	<0.001	0.422	<0.001
>4	0.335	<0.001	0.340	<0.001	0.335	<0.001
**Enabling factor (household head)**						
Educational attainment (up to primary school)						
Junior middle school	0.933	0.058	0.925	0.032	0.926	0.030
Senior middle school	0.935	0.166	0.923	0.098	0.913	0.053
Tertiary education	0.785	0.001	0.778	0.001	0.769	<0.001
Employment (unemployment)						
Employed	0.601	<0.001	0.593	<0.001	0.610	<0.001
**Enabling factor (household)**						
Annual household income per capita (lowest quintile)						
Lower	0.141	<0.001	0.148	<0.001	0.104	<0.001
Higher	0.042	<0.001	0.049	<0.001	0.031	<0.001
Highest quintile	0.024	<0.001	0.028	<0.001	0.017	<0.001
Basic medical insurance (full coverage by UEBMI)						
None	0.812	0.030	0.840	0.065	0.799	0.016
Partial coverage	1.001	0.991	0.992	0.917	0.978	0.770
Full coverage by URRBMI	1.105	0.183	1.098	0.214	1.045	0.558
Full coverage by mixed	0.937	0.419	0.928	0.360	0.93	0.367
commercial medical insurance (no)						
Yes	1.083	0.361	1.005	0.952	1.043	0.623
Age pension for employees (no)						
Yes	0.872	0.014	0.865	0.010	0.879	0.020
Residency (urban)						
Rural	1.075	0.044	1.052	0.158	1.093	0.011
**Need factor**						
Household with elderly (no)						
Yes	1.270	<0.001	1.198	<0.001	1.237	<0.001
Household with children (no)						
Yes	1.05	0.383	1.014	0.798	1.059	0.277
Number of members in poor self-rated health (0)						
1	2.883	<0.001	2.909	<0.001	2.809	<0.001
>1	4.243	<0.001	4.282	<0.001	4.137	<0.001
**Provincial level variable**						
Health expenditure per capita	1.726	0.001	1.567	0.001	1.610	0.001
Percentage of total government expenditure on health	1.015	0.001	1.017	<0.001	1.016	<0.001
Number of hospital beds per 1000 population	1.060	0.375	1.100	0.100	1.073	0.259
Annal hospitalization rate	0.983	0.255	0.976	0.070	0.981	0.169
Average number of medical visits per resident	0.963	0.147	0.983	0.454	0.975	0.293
Model statistics						
LR test (*P*)	0.000		0.000		0.000	
AIC	61 001.914		33 179.624		153 457.674	

*Note:* AOR, adjusted odds ratio; UEBMI, urban employee basic medical insurance; URRBMI, urban and rural resident basic medical insurance; LR test, likelihood ratio test;.AIC, Akaike information criterion.

Higher educational attainment (AOR = 0.778–0.925, *P* < 0.05) and employment (AOR = 0.593–0.601, *P* < 0.001) of household head, larger household size (AOR = 0.335–0.537, *P* < 0.001), and higher household income per capita (AOR = 0.024–0.18, *P* < 0.001) were protective factors against IHE (Table 5).

Older age (AOR = 1.227–1.247, *P* < 0.001) of household head, and households with elderly members (AOR = 1.198–1.270, *P* < 0.001) and more members with self-rated poor health (AOR = 2.883–4.282, *P* < 0.001) were risk factors for IHE (Table 5).

At the provincial level, higher health expenditure per capita (AOR = 1.567–1.726, *P* < 0.01) and a higher governmental share in total health expenditure (AOR = 1.015–1.017, *P* < 0.01) were associated with higher odds of IHE. (Table 5).

The regression models for urban and rural areas (Table S6, see online supplementary material) and different regions (Table S7, see online supplementary material) identified similar predictors of IHE, reinforcing the findings from the national sample.

### Factors associated with CHE and IHE

The regression models on the combined CHE or/and IHE indicators (Table 6) yielded similar results as the models established for each indicator separately. Healthcare-induced poverty increased (AOR = 1.382–1.143 in 2015, *P* < 0.01) prior to the launch of the health poverty alleviation project, before decreasing afterward (AOR = 0.476–0.648 in 2017, *P* < 0.001).

**Table 6.** Factors associated with either CHE or/and IHE: results of two-level logistic regression models

	CHE or IHE		CHE and IHE	
	Model 5		Model 6	
Variable (reference)	AOR	*P*	AOR.	*P*
**Year (2013)**				
2015	1.143	0.001	1.382	<0.001
2017	0.648	<0.001	0.599	<0.001
2019	0.476	<0.001	0.527	<0.001
**Predisposing factor (household head)**				
Gender (female)				
Male	1.026	0.190	0.941	0.112
Age (<45 years)				.
45–54	1.264	<0.001	1.208	0.001
55–65	1.386	<0.001	1.205	0.001
>65	2.086	<0.001	1.601	<0.001
**Predisposing factor (household)**				
Household size (<3)				
3	0.652	<0.001	0.540	<0.001
4	0.588	<0.001	0.412	<0.001
>4	0.528	<0.001	0.354	<0.001
**Enabling factor (household head)**				
Educational attainment (up to primary school)				.
Junior middle school	0.884	<0.001	0.899	0.004
Senior middle school	0.837	<0.001	0.834	<0.001
Tertiary education	0.705	<0.001	0.609	<0.001
Employment (unemployment)				
Employed	0.685	<0.001	0.599	<0.001
**Enabling factor (household)**				
Annual household income per capita (lowest quintile)				
Lower	0.197	<0.001	0.232	<0.001
Higher	0.168	<0.001	0.079	<0.001
Highest quintile	0.158	<0.001	0.050	<0.001
Basic medical insurance (full coverage by UEBMI)				
None	0.953	0.276	0.861	0.131
Partial coverage	0.919	0.012	1.052	0.526
Full coverage by URRBMI	0.903	0.002	1.161	0.055
Full coverage by mixed	0.914	0.008	1.006	0.943
commercial medical insurance (no)				
Yes	0.834	<0.001	0.928	0.455
Age pension for employees (no)				
Yes	1.252	<0.001	0.833	0.001
Residency (urban)				
Rural	1.187	<0.001	1.125	0.001
**Need factor**				
Household with elderly (no)				
Yes	1.307	<0.001	1.323	<0.001
Household with children (no)				
Yes	1.194	<0.001	1.021	0.728
Number of members in poor self-rated health (0)				
1	2.510	<0.001	3.160	<0.001
>1	3.862	<0.001	4.803	<0.001
**Provincial level variable**				
Health expenditure per capita	1.529	0.003	1.377	0.022
Percentage of total government expenditure on health	1.001	0.852	1.017	<0.001
Number of hospital beds per 1000 population	0.899	0.038	1.118	0.057
Annal hospitalization rate	1.019	0.190	0.969	0.018
Average number of medical visits per resident	0.971	0.196	0.988	0.587
Model statistics				
LR test (*P*)	0.000		0.000	
AIC	95 143.513		31 173.933	

*Note:* AOR, adjusted odds ratio; UEBMI, urban employee basic medical insurance;.URRBMI, Urban and rural resident basic medical insurance; LR test, likelihood ratio test; AIC, Akaike information criterion.

Higher educational attainment (AOR = 0.609–0.899, *P* < 0.01) and employment (AOR = 0.599–0.685, *P* < 0.01) of household head, larger household size (AOR = 0.354–0.652, *P* < 0.001), higher household income per capita (AOR = 0.050–0.232, *P* < 0.001), and commercial medical insurance (OR = 0.834, *P* < 0.001) were protective factors against healthcare-induced poverty. Older age of household head (AOR = 1.205–2.086, *P* < 0.01), households with elderly members (AOR = 1.307–1.323, *P* <0.05), rural location (AOR = 1.125–1.187, P < 0.01), and more members with self-rated poor health (AOR = 2.510–4.803, P < 0.001) were risk factors for healthcare-induced poverty (Table 6).

At the provincial level, higher health expenditure per capita (OR = 1.377–1.529, *P* < 0.05) was associated with higher odds of healthcare-induced poverty (Table 6).

The regression models for urban and rural areas (Tables S8 and S9, see online supplementary material) and different regions (Tables S10 and S11, see online supplementary material) identified similar predictors, reinforcing the findings from the national sample.

## Discussion

This study shows that financial hardship due to health expenditure has significantly decreased in China over the years. From 2013 to 2015, there was no significant reduction in CHE and IHE incidence. However, following the launch of the health poverty alleviation project in 2016, the incidence of CHE declined substantially, aligning with findings from previous studies (Chen and J 2019, Lu et al. 2021, Zhang et al. 2022). Despite these achievements, the overall incidence of CHE and IHE in China remains high (21.22% for CHE and 8.66% for IHE in 2019) compared to many other countries. For instance, CHE incidence ranges from 2% to 6% in Japan, 0.5% to 2% in the UK, and 0.8% to 5% in the USA (Wagstaff et al. 2018).

Multiple factors may have contributed to the decline in CHE and IHE. Undeniably, an increase in household income or consumption capacity helps reduce CHE and IHE. In China, annual household income per capita rose from 21 466 in 2013 to 29 140 in 2019. The poverty eradication campaign (State Council 2015) achieved significant success, lowering the proportion of households living below the poverty line from 22.4% in 2013 to 14.2% in 2019. However, income-related inequalities in CHE and IHE persist. Our findings indicate that households in the highest income quartile experienced the most significant reduction in health poverty. Moreover, income and unemployment remain significant predictors of CHE and IHE, even after controlling for variations in other factors, including the year.

The health poverty alleviation project supported poor households by providing greater financial subsidies for healthcare and improving the management of health conditions (State Council 2016c). By 2020, the reimbursement rate of health expenditure from social health insurance for impoverished households had increased by 10% compared to the general population, reaching an actual reimbursement rate of 60% for overall medical expenses and 80% for hospitalizations (NHSA 2020). However, a dilemma arises: lowering financial barriers may drive greater healthcare utilization and increase health expenditure (Wang and Liu 2019). This may help explain the positive association between CHE and IHE and total provincial health expenditure. A key concern is the positive correlation between government health investment and the incidence of IHE observed in this study, suggesting a limited impact of the health poverty alleviation project. This finding aligns with previous reports (Tang et al. 2021). In 2021, the National Healthcare Security Administration incorporated financial protection measures into its assessment of poverty alleviation progress (NHSA 2021). As government health investment continues to grow, more evidence-based and effective strategies are needed to maximize the impact of public funding.

We found that urban households fully covered by UEBMI, the most prestigious social health insurance program, are more likely to incur CHE than others. Empirical evidence suggests that UEBMI enrollees in urban areas have the privilege and convenience of bypassing primary care, often seeking more expensive medical treatment at tertiary hospitals concentrated in metropolitan areas (Yao et al. 2022). China has yet to establish a primary care gatekeeping mechanism (Ye et al. 2024).

Strengthening disease prevention and health management is a key component of the health poverty alleviation project, as poor health is the root cause of health poverty. The findings of this study provide further evidence supporting this argument. We found that older age and poor self-rated health are associated with a higher incidence of CHE and IHE. The Chinese government has sought to reform the hospital-dominated healthcare system by strengthening primary care. Primary care institutions receive funding to systematically manage local residents with chronic conditions (National Health Commission 2017a, State Council 2019c). By early 2019, >98% of patients with chronic and serious health conditions from poor households had received essential treatment (Cheng et al. 2021), largely due to government investment in health infrastructure in impoverished areas, including the establishment of 138 000 rural clinics (Cheng et al. 2021).

Household size, a significant predictor of CHE and IHE, continues to shrink in China, posing a profound challenge to the country’s welfare system. In response, the Chinese government has increasingly relied on insurance tools to address these challenges. Our findings indicate that commercial health insurance is indeed associated with lower odds of healthcare-induced poverty. Policymakers could explore ways to better integrate commercial health insurance into the existing system, enhancing its role in complementing and bridging gaps in basic medical insurance. Some cities have implemented commercial supplementary health insurance schemes to enhance financial protection for impoverished households (Fuzhou 2017, Hangzhou 2020).

Regional inequality remains a significant health policy concern in China. Our findings indicate that regional disparities in CHE and IHE persist, with the central and western regions exhibiting a higher incidence—consistent with previous research (Jia et al. 2023, Wang et al. 2023). Despite the Chinese government’s call to enhance cross-territory collaboration in health poverty alleviation (State Council 2019c), rural and less-developed provinces continue to bear a disproportionate burden of CHE and IHE. This underscores the need for sustained efforts from the central government to address regional disparities.

There are several limitations in this study. Firstly, while the data enabled us to calculate CHE and IHE, it did not capture those who forgo health services due to financial challenges. Secondly, we treated the CHFS 2013–2019 as repeated cross-sectional data without tracking the same households. Caution needs to be taken when inferring a direct causal relationship between the health poverty alleviation project and the outcomes of CHE and IHE due to the lack of a control group. Finally, although the study period (2013–2019) covers both the pre- and post-policy phases of the poverty eradication campaign (2015–2020) and the health poverty alleviation projects (2016–2020), data for 2020 are unavailable.

## Conclusions

China has achieved some success in poverty alleviation campaigns, with household financial hardship due to healthcare expenditure decreasing over time. However, there are disparities between urban and rural areas and across regions, indicating the necessity to focus on equality in future efforts.

## Supplementary Material

czaf026_Supp

## Data Availability

The datasets generated and/or analysed during the current study are publicly available in the China Household Finance Survey (CHFS) repository, https://chfs.swufe.edu.cn/. The China map template was downloaded from Aliyun Data Visualization Lab, http://datav.aliyun.com/portal/school/atlas/area_selector.

## Reflexivity statement

Our author group is inclusive and balanced, consisting of two females and two males. Q.Y., an associate professor in the School of Political Science and Public Administration, brings expertise in health economics and health insurance. C.L., a professor in the School of Psychology and Public Health, specializes in health equity. The other two members hold master’s degrees in public health and public administration, respectively. Our team’s diverse educational backgrounds, spanning mainland China, Hong Kong SAR, Australia, and the UK, contribute to the wide range of perspectives in our research.

## List of abbreviations

AIC: Akaike Information Criterion

AOR: Adjusted odds ratio

CHE: Catastrophic health expenditure

CHFS: China Household Finance Survey

IHE: Impoverishment health expenditure

NHSA: National Healthcare Security Administration

MOHRRS: Ministry of Human Resources and Social Security

SDGs: Sustainable development goals

UEBMI: Urban Employee Basic Medical Insurance

UN: United Nations

URRBMI: Urban and Rural Resident Basic Medical Insurance

WHO: World Health Organization
