# Supplementary material for: Trends and determinants of healthcare-induced poverty in China 2013–2019
Source: Health Policy Plan. 2025 Jun 12;40(6):625–40. doi: 10.1093/heapol/czaf026 (PMC12160802; doi:10.1093/heapol/czaf026)
Supplement: czaf026_Supp [file czaf026_supp.zip › Supplementary appendix (correction).docx]

**Supplementary Appendix**

# Table S1: Measurements of total income, total expenditures, and health expenditures

| Indicator | Measurement and Source |
| --- | --- |
| Total income | Total household income = Wage income + Agricultural income + Business income + Property income + Transfer income (e.g., insurance compensation. housing provident fund, and private transfer income)  See details in Chapter 1.3 (2013), Chapter 1.4 (2015-207), Chapter 1.7 (2019), and Chapters 4.4–4.5 of the survey questionnaire (available at: https://chfs.swufe.edu.cn/). |
| Total expenditure | Total household expenditure = Food expenditure + Clothing expenditure + Housing expenditure + Household equipment and services expenditure + Transportation and communication expenditure + Education and entertainment expenditure + Health expenditure + Other expenditures  See details in Chapters 4.1–4.3 (2013-2019) of the questionnaire (available at: https://chfs.swufe.edu.cn/). |
| Health expenditure | Out-of-pocket health expenditure = Total health expenditure – health insurance reimbursements  2013: [F2019] How much was spent on health expenditures last year?  [F2020] How much of this was covered by health insurance?  In the 2015 and 2017 surveys, the same questions were included with updated question numbers: [G1019] and [G1019a]  2019: [F2024] How much was the expenditure on hospitalization?  [F2025] How much of this was covered by health insurance?  [F2028] How much was the expenditure on non-hospitalization medical care?  [F2029] How much of this was covered by health insurance? |

**Table S2: Incidence of catastrophic health expenditure (CHE) and impoverishing health expenditure (IHE) by household characteristics (income and health status) from 2013 to 2019**

| Household group | Indicator | 2013 | 2015 | 2017 | 2019 | Annual average growth rate |
| --- | --- | --- | --- | --- | --- | --- |
| Lowest income | CHE | 38.82% | 34.44% | 29.37% | 30.26% | -4.07% |
|  | IHE | 58.22% | 60.64% | 29.86% | 43.53% | -4.73% |
|  | CHE or IHE | 79.16% | 88.91% | 51.30% | 62.82% | -3.78% |
|  | CHE and IHE | 41.02% | 29.38% | 21.06% | 24.69% | -8.11% |
| Lower income | CHE | 33.48% | 26.92% | 24.94% | 23.46% | -5.76% |
|  | IHE | 8.13% | 10.73% | 6.06% | 6.86% | -2.79% |
|  | CHE or IHE | 33.92% | 28.27% | 25.07% | 24.15% | -5.50% |
|  | CHE and IHE | 7.69% | 9.37% | 5.93% | 6.18% | -3.58% |
| Higher income | CHE | 30.00% | 22.91% | 22.31% | 18.68% | -7.59% |
|  | IHE | 2.77% | 2.86% | 2.15% | 2.26% | -3.33% |
|  | CHE or IHE | 30.07% | 22.93% | 22.37% | 18.74% | -7.58% |
|  | CHE and IHE | 2.69% | 2.84% | 2.09% | 2.21% | -3.22% |
| Highest income | CHE | 24.99% | 19.99% | 24.59% | 12.46% | -10.95% |
|  | IHE | 1.41% | 1.37% | 1.72% | 0.75% | -9.99% |
|  | CHE or IHE | 25.02% | 19.99% | 24.61% | 12.49% | -10.93% |
|  | CHE and IHE | 1.38% | 1.37% | 1.70% | 0.72% | -10.28% |
| All members in good health | CHE | 15.46% | 18.88% | 16.49% | 12.22% | -3.84% |
|  | IHE | 3.25% | 4.13% | 3.34% | 4.16% | 4.20% |
|  | CHE or IHE | 18.56% | 23.08% | 18.73% | 15.18% | -3.29% |
|  | CHE and IHE | 2.03% | 2.74% | 2.38% | 2.32% | 2.25% |
| One member in poor self-rated health | CHE | 35.93% | 39.48% | 39.08% | 33.60% | -1.11% |
|  | IHE | 10.59% | 14.41% | 13.60% | 15.37% | 6.41% |
|  | CHE or IHE | 42.40% | 49.02% | 45.17% | 41.41% | -0.39% |
|  | CHE and IHE | 8.58% | 11.78% | 11.58% | 11.16% | 4.48% |
| More than one member in poor self-rated health | CHE | 45.07% | 49.34% | 50.13% | 45.93% | 0.32% |
|  | IHE | 15.58% | 21.34% | 21.70% | 23.25% | 6.90% |
|  | CHE or IHE | 52.63% | 62.59% | 60.01% | 56.95% | 1.32% |
|  | CHE and IHE | 13.05% | 17.59% | 18.60% | 17.82% | 5.33% |

**Table S3: Incidence of catastrophic health expenditure (CHE) and impoverishing health expenditure (IHE) by rural/urban locations and regions from 2013 to 2019**

| Indicators | Area | 2013 | 2015 | 2017 | 2019 | Average annual growth rate |
| --- | --- | --- | --- | --- | --- | --- |
| CHE | Urban | 28.56% | 23.73% | 23.21% | 18.71% | -6.81% |
|  | Rural | 38.85% | 31.20% | 29.78% | 25.75% | -6.62% |
| IHE | Urban | 6.53% | 5.62% | 4.79% | 5.88% | -1.73% |
|  | Rural | 17.38% | 12.95% | 13.90% | 14.52% | -2.95% |
| CHE or IHE | Urban | 32.53% | 27.41% | 25.42% | 21.63% | -6.58% |
|  | Rural | 49.15% | 43.99% | 37.42% | 34.82% | -5.58% |
| CHE and IHE | Urban | 5.16% | 4.44% | 3.89% | 4.24% | -3.22% |
|  | Rural | 14.12% | 9.22% | 11.21% | 9.47% | -6.44% |
| CHE | Eastern | 30.94% | 25.37% | 23.73% | 18.39% | -8.31% |
|  | Central | 32.62% | 27.10% | 26.77% | 23.55% | -5.29% |
|  | Western | 32.54% | 26.41% | 27.01% | 22.89% | -5.69% |
| IHE | Eastern | 7.27% | 5.94% | 5.41% | 6.72% | -1.30% |
|  | Central | 11.15% | 8.76% | 8.95% | 10.09% | -1.65% |
|  | Western | 12.37% | 9.41% | 10.12% | 10.09% | -6.81% |
| CHE or IHE | Eastern | 35.38% | 30.01% | 26.21% | 21.88% | -7.70% |
|  | Central | 39.23% | 33.77% | 31.24% | 28.84% | -5.00% |
|  | Western | 39.60% | 34.54% | 32.59% | 29.32% | -4.89% |
| CHE and IHE | Eastern | 5.94% | 4.61% | 4.52% | 4.78% | -3.56% |
|  | Central | 8.79% | 6.74% | 7.25% | 6.96% | -3.82% |
|  | Western | 9.87% | 6.71% | 7.87% | 6.58% | -6.53% |

# Table S4: Factors associated with the incidence of catastrophic health expenditure (CHE) in rural and urban areas

| Variables (Reference) | Urban areas | | Rural areas | |
| --- | --- | --- | --- | --- |
|  | AOR | p | AOR | p |
| **Year (2013)** |  |  |  | . |
| 2015 | 1.090 | 0.005 | 0.887 | 0.006 |
| 2017 | 0.831 | <0.001 | 0.647 | <0.001 |
| 2019 | 0.545 | <0.001 | 0.339 | 0.013 |
| **Predisposing factors (household head)** |  |  |  |  |
| Gender (Female) |  | . |  | . |
| Male | 1.027 | 0.206 | 0.935 | 0.086 |
| Age (<45 years) |  |  |  |  |
| 45-54 | 1.378 | <0.001 | 1.157 | <0.001 |
| 55-65 | 1.508 | <0.001 | 1.362 | <0.001 |
| >65 | 2.531 | <0.001 | 1.674 | <0.001 |
| **Predisposing factors (household)** |  |  |  |  |
| Household size (<3) |  |  |  |  |
| 3 | 0.678 | <0.001 | 0.701 | <0.001 |
| 4 | 0.608 | <0.001 | 0.618 | <0.001 |
| >4 | 0.562 | <0.001 | 0.569 | <0.001 |
| **Enabling factors (household head)** |  |  |  |  |
| Educational attainment (Up to primary school) |  |  |  | . |
| Junior middle school | 0.891 | <0.001 | 0.876 | <0.001 |
| Senior middle school | 0.829 | <0.001 | 0.854 | 0.001 |
| Tertiary education | 0.707 | <0.001 | 0.855 | 0.232 |
| Employment (Unemployment) |  |  |  |  |
| Employed | 0.737 | <0.001 | 0.676 | <0.001 |
| **Enabling factors (household)** |  |  |  |  |
| Annual household income per capita (Lowest quintile) |  |  |  |  |
| Lower | 0.945 | 0.059 | 0.946 | 0.053 |
| Higher | 0.845 | <0.001 | 0.878 | 0.001 |
| Highest quintile | 0.820 | <0.001 | 0.780 | <0.001 |
| Basic medical insurance (Full coverage of UEBMI) |  | . |  |  |
| None | 0.945 | 0.229 | 1.207 | 0.477 |
| Partial coverage | 0.933 | 0.052 | 1.234 | 0.421 |
| Full coverage of URRBMI | 0.978 | 0.52 | 1.251 | 0.388 |
| Full coverage of mixed | 0.949 | 0.132 | 1.336 | 0.273 |
| Commercial medical insurance (No) |  |  |  |  |
| Yes | 0.840 | <0.001 | 0.725 | 0.001 |
| Age pension for employees (No) |  |  |  |  |
| Yes | 1.246 | <0.001 | 0.854 | 0.043 |
| **Need factors** |  |  |  |  |
| Household with elderly (No) |  |  |  |  |
| Yes | 1.352 | <0.001 | 1.305 | <0.001 |
| Household with children (No) |  |  |  |  |
| Yes | 1.219 | <0.001 | 1.226 | <0.001 |
| Number of members in poor self-rated health (0) |  |  |  |  |
| 1 | 2.570 | <0.001 | 2.274 | <0.001 |
| >1 | 3.959 | <0.001 | 3.640 | <0.001 |
| **Provincial level variables** |  |  |  |  |
| Health expenditure per capita | 1.472 | <0.001 | 1.228 | 0.045 |
| Percentage of total government expenditure on health | 1.002 | 0.244 | 1.011 | <0.001 |
| Number of hospital beds per 1 000 population | 0.944 | 0.056 | 1.088 | 0.027 |
| Annal hospitalization rate | 0.992 | 0.247 | 0.980 | 0.015 |
| Average number of medical visits per resident | 0.964 | 0.001 | 1.014 | 0.344 |

Note: AOR-adjusted odds ratio; UEBMI-urban employee basic medical insurance; URRBMI-urban and rural resident basic medical insurance

# Table S5: Factors associated with the incidence of catastrophic health expenditure (CHE) in eastern developed, central developing, and western underdeveloped regions

| Variables (Reference) | Eastern Region | | Central Region | | West Region | |
| --- | --- | --- | --- | --- | --- | --- |
|  | AOR | p | AOR | p | AOR | p |
| **Year (2013)** |  |  |  |  |  | . |
| 2015 | 1.06 | 0.120 | 1.107 | 0.069 | 0.953 | 0.416 |
| 2017 | 0.772 | <0.001 | 0.889 | 0.196 | 0.823 | 0.029 |
| 2019 | 0.446 | <0.001 | 0.710 | 0.026 | 0.582 | <0.001 |
| **Predisposing factors (household head)** |  |  |  |  |  |  |
| Gender (Female) |  |  |  | . |  |  |
| Male | 0.992 | 0.769 | 1.022 | 0.556 | 1.067 | 0.091 |
| Age (<45 years) |  |  |  |  |  |  |
| 45-54 | 1.372 | <0.001 | 1.308 | <0.001 | 1.211 | <0.001 |
| 55-65 | 1.456 | <0.001 | 1.530 | <0.001 | 1.358 | <0.001 |
| >65 | 2.284 | <0.001 | 2.140 | <0.001 | 2.028 | <0.001 |
| **Predisposing factors (household)** |  |  |  |  |  |  |
| Household size (<3) |  |  |  |  |  |  |
| 3 | 0.701 | <0.001 | 0.610 | <0.001 | 0.722 | <0.001 |
| 4 | 0.682 | <0.001 | 0.533 | <0.001 | 0.612 | <0.001 |
| >4 | 0.605 | <0.001 | 0.512 | <0.001 | 0.571 | <0.001 |
| **Enabling factors (household head)** |  |  |  |  |  |  |
| Educational attainment (Up to primary school) |  |  |  |  |  |  |
| Junior middle school | 0.906 | 0.001 | 0.866 | <0.001 | 0.853 | <0.001 |
| Senior middle school | 0.802 | <0.001 | 0.853 | <0.001 | 0.816 | <0.001 |
| Tertiary education | 0.706 | <0.001 | 0.687 | <0.001 | 0.646 | <0.001 |
| Employment (Unemployment) |  |  |  |  |  |  |
| Employed | 0.689 | <0.001 | 0.703 | <0.001 | 0.757 | <0.001 |
| **Enabling factors (household)** |  |  |  |  |  |  |
| Annual household income per capita (Lowest quintile) |  |  |  | . |  | . |
| Lower | 0.932 | 0.035 | 0.995 | 0.889 | 0.923 | 0.038 |
| Higher | 0.838 | <0.001 | 0.919 | 0.053 | 0.813 | <0.001 |
| Highest quintile | 0.798 | <0.001 | 0.926 | 0.161 | 0.785 | <0.001 |
| Basic medical insurance (Full coverage of UEBMI) |  |  |  |  |  |  |
| None | 0.857 | 0.005 | 0.923 | 0.352 | 1.107 | 0.287 |
| Partial coverage | 0.882 | 0.004 | 0.973 | 0.687 | 1.016 | 0.839 |
| Full coverage of URRBMI | 0.913 | 0.030 | 0.948 | 0.417 | 1.068 | 0.396 |
| Full coverage of mixed | 0.929 | 0.085 | 0.913 | 0.194 | 1.046 | 0.573 |
| Commercial medical insurance (No) |  |  |  |  |  |  |
| Yes | 0.850 | 0.002 | 0.684 | <0.001 | 0.843 | 0.061 |
| Age pension for employees (No) |  |  |  |  |  |  |
| Yes | 1.177 | <0.001 | 1.215 | <0.001 | 1.492 | <0.001 |
| Residency (Urban) |  |  |  |  |  |  |
| Rural | 1.107 | 0.001 | 1.237 | <0.001 | 1.314 | <0.001 |
| **Need factors** |  |  |  |  |  |  |
| Household with elderly (No) |  |  |  |  |  |  |
| Yes | 1.364 | <0.001 | 1.359 | <0.001 | 1.263 | <0.001 |
| Household with children (No) |  |  |  |  |  |  |
| Yes | 1.191 | <0.001 | 1.210 | <0.001 | 1.251 | <0.001 |
| Number of members in poor self-rated health (0) |  |  |  |  |  |  |
| 1 | 2.469 | <0.001 | 2.550 | <0.001 | 2.327 | <0.001 |
| >1 | 3.659 | <0.001 | 4.057 | <0.001 | 3.795 | <0.001 |
| **Provincial level variables** |  |  |  |  |  |  |
| Health expenditure per capita | 1.373 | 0.002 | 1.233 | 0.215 | 1.646 | <0.001 |
| Percentage of total government expenditure on health | 1.000 | 0.914 | 0.995 | 0.39 | 0.997 | 0.453 |
| Number of hospital beds per 1 000 population | 0.993 | 0.876 | 0.921 | 0.163 | 0.902 | 0.053 |
| Annal hospitalization rate | 0.974 | 0.054 | 0.982 | 0.032 | 0.995 | 0.658 |
| Average number of medical visits per resident | 0.965 | 0.043 | 1.079 | <0.001 | 0.867 | <0.001 |

Note: AOR-adjusted odds ratio; UEBMI-urban employee basic medical insurance; URRBMI-urban and rural resident basic medical insurance

# Table S6: Factors associated with the incidence of impoverished health expenditure (IHE) in rural and urban areas

| Variables (Reference) | Urban areas | | Rural areas | |
| --- | --- | --- | --- | --- |
|  | AOR | p | AOR | p |
| **Year (2013)** |  |  |  |  |
| 2015 | 1.636 | <0.001 | 1.550 | <0.001 |
| 2017 | 0.525 | <0.001 | 0.416 | <0.001 |
| 2019 | 0.684 | 0.007 | 0.276 | 0.104 |
| **Predisposing factors (household head)** |  |  |  |  |
| Gender (Female) |  |  |  | . |
| Male | 0.956 | 0.310 | 0.929 | 0.282 |
| Age (<45 years) |  | . |  |  |
| 45-54 | 1.079 | 0.233 | 0.969 | 0.664 |
| 55-65 | 1.062 | 0.401 | 0.929 | 0.328 |
| >65 | 1.435 | <0.001 | 1.055 | 0.538 |
| **Predisposing factors (household)** |  |  |  |  |
| Household size (<3) |  |  |  |  |
| 3 | 0.512 | <0.001 | 0.570 | <0.001 |
| 4 | 0.403 | <0.001 | 0.430 | <0.001 |
| >4 | 0.331 | <0.001 | .326 | <0.001 |
| **Enabling factors (household head)** |  |  |  |  |
| Educational attainment (Up to primary school) |  |  |  |  |
| Junior middle school | 0.969 | 0.528 | 0.905 | 0.050 |
| Senior middle school | 0.984 | 0.792 | 0.890 | 0.162 |
| Tertiary education | 0.882 | 0.115 | 0.505 | 0.039 |
| Employment (Unemployment) |  |  |  |  |
| Employed | 0.611 | <0.001 | 0.609 | <0.001 |
| **Enabling factors (household)** |  |  |  |  |
| Annual household income per capita (Lowest quintile) |  |  |  |  |
| Lower | 0.104 | <0.001 | 0.103 | <0.001 |
| Higher | 0.030 | <0.001 | 0.031 | <0.001 |
| Highest quintile | 0.016 | <0.001 | 0.028 | <0.001 |
| Basic medical insurance (Full coverage of UEBMI) |  |  |  |  |
| None | 0.812 | 0.048 | 0.517 | 0.163 |
| Partial coverage | 0.965 | 0.666 | 0.645 | 0.344 |
| Full coverage of URRBMI | 1.126 | 0.138 | 0.635 | 0.325 |
| Full coverage of mixed | 0.946 | 0.510 | 0.607 | 0.294 |
| Commercial medical insurance (No) |  |  |  |  |
| Yes | 1.104 | 0.324 | 0.974 | 0.878 |
| Age pension for employees (No) |  |  |  |  |
| Yes | 0.853 | 0.013 | 0.807 | 0.149 |
| **Need factors** |  |  |  |  |
| Household with elderly (No) |  |  |  |  |
| Yes | 1.280 | <0.001 | 1.191 | 0.005 |
| Household with children (No) |  |  |  |  |
| Yes | 1.082 | 0.286 | 1.056 | 0.479 |
| Number of members in poor self-rated health (0) |  |  |  |  |
| 1 | 3.130 | <0.001 | 2.417 | <0.001 |
| >1 | 4.177 | <0.001 | 4.008 | <0.001 |
| **Provincial level variables** |  |  |  |  |
| Health expenditure per capita | 1.499 | 0.005 | 1.531 | 0.015 |
| Percentage of total government expenditure on health | 1.008 | 0.056 | 1.025 | <0.001 |
| Number of hospital beds per 1 000 population | 1.048 | 0.450 | 1.176 | 0.017 |
| Annal hospitalization rate | 0.979 | 0.139 | 0.969 | 0.033 |
| Average number of medical visits per resident | 0.984 | 0.485 | 0.990 | 0.700 |

Note: AOR-adjusted odds ratio; UEBMI-urban employee basic medical insurance; URRBMI-urban and rural resident basic medical insurance

# Table S7: Factors associated with the incidence of impoverished health expenditure (IHE) in eastern developed, central developing, and western underdeveloped regions

| Variables (Reference) | Eastern Region | | Central Region | | West Region | |
| --- | --- | --- | --- | --- | --- | --- |
|  | AOR | p | AOR | p | AOR | p |
| **Year (2013)** |  | . |  | . |  |  |
| 2015 | 1.673 | <0.001 | 1.515 | <0.001 | 1.573 | <0.001 |
| 2017 | 0.584 | <0.001 | 0.366 | <0.001 | 0.475 | <0.001 |
| 2019 | 0.595 | 0.021 | 0.680 | 0.153 | 0.631 | 0.086 |
| **Predisposing factors (household head)** |  |  |  |  |  |  |
| Gender (Female) |  |  |  | . |  |  |
| Male | 0.966 | 0.532 | 0.991 | 0.898 | 0.911 | 0.186 |
| Age (<45 years) |  | . |  | . |  | . |
| 45-54 | 1.076 | 0.354 | 1.105 | 0.251 | 0.971 | 0.721 |
| 55-65 | 1.011 | 0.894 | 1.096 | 0.327 | 0.954 | 0.611 |
| >65 | 1.297 | 0.007 | 1.241 | 0.046 | 1.242 | 0.044 |
| **Predisposing factors (household)** |  |  |  |  |  |  |
| Household size (<3) |  |  |  |  |  |  |
| 3 | 0.564 | <0.001 | 0.485 | <0.001 | 0.539 | <0.001 |
| 4 | 0.431 | <0.001 | 0.352 | <0.001 | 0.473 | <0.001 |
| >4 | 0.342 | <0.001 | 0.313 | <0.001 | 0.341 | <0.001 |
| **Enabling factors (household head)** |  |  |  |  |  |  |
| Educational attainment (Up to primary school) |  | . |  | . |  | . |
| Junior middle school | 0.915 | 0.118 | 0.951 | 0.421 | 0.957 | 0.512 |
| Senior middle school | 0.932 | 0.323 | 0.868 | 0.098 | 0.952 | 0.605 |
| Tertiary education | 0.851 | 0.110 | 0.777 | 0.082 | 0.657 | 0.008 |
| Employment (Unemployment) |  | . |  | . |  | . |
| Employed | 0.596 | <0.001 | 0.588 | <0.001 | 0.661 | <0.001 |
| **Enabling factors (household)** |  |  |  |  |  |  |
| Annual household income per capita (Lowest quintile) |  |  |  |  |  | . |
| Lower | 0.115 | <0.001 | 0.094 | <0.001 | 0.100 | <0.001 |
| Higher | 0.035 | <0.001 | 0.028 | <0.001 | 0.026 | <0.001 |
| Highest quintile | 0.017 | <0.001 | 0.020 | <0.001 | 0.017 | <0.001 |
| Basic medical insurance (Full coverage of UEBMI) |  | . |  | . |  | . |
| None | 0.609 | <0.001 | 0.939 | 0.732 | 1.317 | 0.215 |
| Partial coverage | 0.920 | 0.418 | 0.987 | 0.932 | 1.269 | 0.216 |
| Full coverage of URRBMI | 0.918 | 0.392 | 1.060 | 0.697 | 1.466 | 0.043 |
| Full coverage of mixed | 0.921 | 0.436 | 0.884 | 0.450 | 1.124 | 0.559 |
| Commercial medical insurance (No) |  | . |  | . |  | . |
| Yes | 1.304 | 0.022 | 0.994 | 0.970 | 0.649 | 0.037 |
| Age pension for employees (No) |  | . |  | . |  |  |
| Yes | 0.757 | 0.001 | 0.805 | 0.034 | 1.332 | 0.015 |
| Residency (Urban) |  | . |  | . |  |  |
| Rural | 0.959 | 0.455 | 1.077 | 0.240 | 1.303 | <0.001 |
| **Need factors** |  |  |  |  |  |  |
| Household with elderly (No) |  |  |  |  |  |  |
| Yes | 1.394 | <0.001 | 1.291 | 0.001 | 1.032 | 0.689 |
| Household with children (No) |  |  |  |  |  |  |
| Yes | 0.912 | 0.304 | 1.121 | 0.234 | 1.170 | 0.088 |
| Number of members in poor self-rated health (0) |  |  |  | . |  | . |
| 1 | 3.019 | <0.001 | 2.903 | <0.001 | 2.382 | <0.001 |
| >1 | 4.146 | <0.001 | 4.062 | <0.001 | 4.095 | <0.001 |
| **Provincial level variables** |  |  |  |  |  |  |
| Health expenditure per capita | 1.263 | 0.278 | 1.620 | 0.114 | 1.534 | 0.082 |
| Percentage of total government expenditure on health | 1.010 | 0.249 | 1.010 | 0.344 | 1.016 | 0.027 |
| Number of hospital beds per 1 000 population | 1.040 | 0.673 | 1.179 | 0.131 | 1.099 | 0.345 |
| Annal hospitalization rate | 0.984 | 0.560 | 0.984 | 0.306 | 0.948 | 0.005 |
| Average number of medical visits per resident | 1.006 | 0.866 | 0.958 | 0.228 | 0.996 | 0.953 |

Note: AOR-adjusted odds ratio; UEBMI-urban employee basic medical insurance; URRBMI-urban and rural resident basic medical insurance

# Table S8: Factors associated with the incidence of catastrophic health expenditure (CHE) or impoverishing health expenditure (IHE) in rural and urban areas

| Variables (Reference) | Urban areas | | Rural areas | |
| --- | --- | --- | --- | --- |
|  | AOR | p | AOR. | p |
| **Year (2013)** |  |  |  | . |
| 2015 | 1.150 | <0.001 | 1.166 | 0.002 |
| 2017 | 0.731 | <0.001 | 0.517 | <0.001 |
| 2019 | 0.525 | <0.001 | 0.281 | 0.004 |
| **Predisposing factors (household head)** |  |  |  |  |
| Gender (Female) |  |  |  | . |
| Male | 1.030 | 0.190 | 0.938 | 0.165 |
| Age (<45 years) |  |  |  | . |
| 45-54 | 1.329 | <0.001 | 1.112 | 0.017 |
| 55-65 | 1.444 | <0.001 | 1.294 | <0.001 |
| >65 | 2.378 | <0.001 | 1.592 | <0.001 |
| **Predisposing factors (household)** |  |  |  |  |
| Household size (<3) |  |  |  |  |
| 3 | 0.648 | <0.001 | 0.669 | <0.001 |
| 4 | 0.569 | <0.001 | 0.585 | <0.001 |
| >4 | 0.505 | <0.001 | 0.526 | <0.001 |
| **Enabling factors (household head)** |  |  |  |  |
| Educational attainment (Up to primary school) | 0.917 | 0.002 | 0.853 | <0.001 |
| Junior middle school | 0.867 | <0.001 | 0.852 | 0.001 |
| Senior middle school | 0.750 | <0.001 | 0.856 | 0.255 |
| Tertiary education |  |  |  |  |
| Employment (Unemployment) | 0.709 | <0.001 | 0.663 | <0.001 |
| Employed |  |  |  |  |
| **Enabling factors (household)** |  |  |  |  |
| Annual household income per capita (Lowest quintile) | 0.182 | <0.001 | 0.206 | <0.001 |
| Lower | 0.152 | <0.001 | 0.184 | <0.001 |
| Higher | 0.143 | <0.001 | 0.166 | <0.001 |
| Highest quintile |  | . |  | . |
| Basic medical insurance (Full coverage of UEBMI) | 0.976 | 0.625 | 1.16 | 0.594 |
| None | 0.939 | 0.080 | 1.081 | 0.774 |
| Partial coverage | 0.953 | 0.181 | 1.021 | 0.938 |
| Full coverage of URRBMI | 0.945 | 0.106 | 1.171 | 0.566 |
| Full coverage of mixed |  |  |  | . |
| Commercial medical insurance (No) | 0.877 | 0.003 | 0.760 | 0.007 |
| Yes |  |  |  | . |
| Age pension for employees (No) | 1.240 | <0.001 | 0.850 | 0.046 |
| Yes |  |  |  |  |
| **Need factors** |  |  |  |  |
| Household with elderly (No) | 1.339 | <0.001 | 1.262 | <0.001 |
| Yes |  |  |  |  |
| Household with children (No) | 1.215 | <0.001 | 1.195 | <0.001 |
| Yes |  |  |  |  |
| Number of members in poor self-rated health (0) | 2.597 | <0.001 | 2.346 | <0.001 |
| 1 | 3.890 | <0.001 | 3.792 | <0.001 |
| >1 |  |  |  |  |
| **Provincial level variables** | 1.564 | <0.001 | 1.478 | 0.001 |
| Health expenditure per capita | 1.003 | 0.254 | 1.018 | <0.001 |
| Percentage of total government expenditure on health | 0.927 | 0.016 | 1.034 | 0.448 |
| Number of hospital beds per 1 000 population | 0.996 | 0.637 | 0.986 | 0.148 |
| Annal hospitalization rate | 0.962 | 0.001 | 0.997 | 0.861 |

Note: AOR-adjusted odds ratio; UEBMI-urban employee basic medical insurance; URRBMI-urban and rural resident basic medical insurance

# Table S9: Factors associated with the incidence of catastrophic health expenditure (CHE) and impoverishing health expenditure (IHE) in rural and urban areas

| Variables (Reference) | Urban areas | | Rural areas | |
| --- | --- | --- | --- | --- |
|  | AOR | p | AOR. | p |
| **Year (2013)** |  | . |  | . |
| 2015 | 1.558 | <0.001 | 1.181 | 0.040 |
| 2017 | 0.669 | <0.001 | 0.525 | <0.001 |
| 2019 | 0.592 | 0.001 | 0.214 | 0.141 |
| **Predisposing factors (household head)** |  |  |  |  |
| Gender (Female) |  | . |  | . |
| Male | 0.941 | 0.197 | 0.900 | 0.128 |
| Age (<45 years) |  | . |  |  |
| 45-54 | 1.313 | <0.001 | 1.064 | 0.449 |
| 55-65 | 1.353 | <0.001 | 1.046 | 0.590 |
| >65 | 2.017 | <0.001 | 1.223 | 0.031 |
| **Predisposing factors (household)** |  |  |  |  |
| Household size (<3) |  |  |  |  |
| 3 | 0.534 | <0.001 | 0.566 | <0.001 |
| 4 | 0.409 | <0.001 | 0.410 | <0.001 |
| >4 | 0.374 | <0.001 | 0.332 | <0.001 |
| **Enabling factors (household head)** |  |  |  |  |
| Educational attainment (Up to primary school) |  |  |  |  |
| Junior middle school | 0.952 | 0.342 | 0.873 | 0.011 |
| Senior middle school | 0.909 | 0.132 | 0.803 | 0.017 |
| Tertiary education | 0.713 | <0.001 | 0.287 | 0.007 |
| Employment (Unemployment) |  |  |  |  |
| Employed | 0.604 | <0.001 | 0.590 | <0.001 |
| **Enabling factors (household)** |  |  |  |  |
| Annual household income per capita (Lowest quintile) |  |  |  |  |
| Lower | 0.255 | <0.001 | 0.217 | <0.001 |
| Higher | 0.085 | <0.001 | 0.073 | <0.001 |
| Highest quintile | 0.050 | <0.001 | 0.066 | <0.001 |
| Basic medical insurance (Full coverage of UEBMI) |  |  |  |  |
| None | 0.887 | 0.287 | 0.710 | 0.512 |
| Partial coverage | 1.025 | 0.773 | 0.888 | 0.816 |
| Full coverage of URRBMI | 1.254 | 0.007 | 0.917 | 0.865 |
| Full coverage of mixed | 1.005 | 0.957 | 0.874 | 0.797 |
| Commercial medical insurance (No) |  |  |  | . |
| Yes | 0.971 | 0.801 | 0.894 | 0.562 |
| Age pension for employees (No) |  |  |  |  |
| Yes | 0.812 | 0.002 | 0.725 | 0.038 |
| **Need factors** |  |  |  |  |
| Household with elderly (No) |  |  |  |  |
| Yes | 1.290 | <0.001 | 1.338 | <0.001 |
| Household with children (No) |  |  |  |  |
| Yes | 1.062 | 0.468 | 1.001 | 0.993 |
| Number of members in poor self-rated health (0) |  |  |  |  |
| 1 | 3.608 | <0.001 | 2.624 | <0.001 |
| >1 | 4.99 | <0.001 | 4.473 | <0.001 |
| **Provincial level variables** |  |  |  |  |
| Health expenditure per capita | 1.431 | 0.020 | 1.264 | 0.199 |
| Percentage of total government expenditure on health | 1.009 | 0.053 | 1.025 | <0.001 |
| Number of hospital beds per 1 000 population | 1.05 | 0.461 | 1.236 | 0.003 |
| Annal hospitalization rate | 0.978 | 0.148 | 0.951 | 0.001 |
| Average number of medical visits per resident | 0.974 | 0.294 | 1.021 | 0.444 |

Note: AOR-adjusted odds ratio; UEBMI-urban employee basic medical insurance; URRBMI-urban and rural resident basic medical insurance

# Table S10: Factors associated with the incidence of catastrophic health expenditure (CHE) or impoverishing health expenditure (IHE) in eastern developed, central developing, and western underdeveloped regions

| Variables (Reference) | Eastern Region | | Central Region | | West Region | |
| --- | --- | --- | --- | --- | --- | --- |
|  | AOR | p | AOR | p | AOR | p |
| **Year (2013)** |  | . |  | . |  | . |
| 2015 | 1.155 | <0.001 | 1.197 | 0.003 | 1.232 | 0.002 |
| 2017 | 0.686 | <0.001 | 0.674 | <0.001 | 0.739 | 0.002 |
| 2019 | 0.436 | <0.001 | 0.619 | 0.003 | 0.630 | 0.004 |
| **Predisposing factors (household head)** |  |  |  |  |  |  |
| Gender (Female) |  | . |  | . |  | . |
| Male | 1.005 | 0.859 | 1.030 | 0.452 | 1.067 | 0.120 |
| Age (<45 years) |  |  |  |  |  | . |
| 45-54 | 1.352 | <0.001 | 1.249 | <0.001 | 1.142 | 0.006 |
| 55-65 | 1.405 | <0.001 | 1.464 | <0.001 | 1.256 | <0.001 |
| >65 | 2.197 | <0.001 | 2.025 | <0.001 | 1.945 | <0.001 |
| **Predisposing factors (household)** |  |  |  |  |  |  |
| Household size (<3) |  |  |  |  |  |  |
| 3 | 0.685 | <0.001 | 0.573 | <0.001 | 0.666 | <0.001 |
| 4 | 0.657 | <0.001 | 0.493 | <0.001 | 0.557 | <0.001 |
| >4 | 0.580 | <0.001 | 0.457 | <0.001 | 0.489 | <0.001 |
| **Enabling factors (household head)** |  |  |  |  |  |  |
| Educational attainment (Up to primary school) |  |  |  |  |  |  |
| Junior middle school | 0.905 | 0.001 | 0.856 | <0.001 | 0.889 | 0.005 |
| Senior middle school | 0.808 | <0.001 | 0.876 | 0.005 | 0.860 | 0.005 |
| Tertiary education | 0.725 | <0.001 | 0.714 | <0.001 | 0.679 | <0.001 |
| Employment (Unemployment) |  |  |  |  |  |  |
| Employed | 0.669 | <0.001 | 0.689 | <0.001 | 0.723 | <0.001 |
| **Enabling factors (household)** |  |  |  |  |  |  |
| Annual household income per capita (Lowest quintile) |  |  |  |  |  |  |
| Lower | 0.191 | <0.001 | 0.211 | <0.001 | 0.189 | <0.001 |
| Higher | 0.163 | <0.001 | 0.183 | <0.001 | 0.156 | <0.001 |
| Highest quintile | 0.150 | <0.001 | 0.177 | <0.001 | 0.146 | <0.001 |
| Basic medical insurance (Full coverage of UEBMI) |  |  |  |  |  | . |
| None | 0.903 | 0.082 | 0.959 | 0.655 | 1.152 | 0.171 |
| Partial coverage | 0.879 | 0.003 | 0.944 | 0.417 | 1.049 | 0.562 |
| Full coverage of URRBMI | 0.868 | 0.001 | 0.910 | 0.166 | 1.012 | 0.885 |
| Full coverage of mixed | 0.911 | 0.033 | 0.899 | 0.136 | 1.050 | 0.552 |
| Commercial medical insurance (No) |  | . |  | . |  | . |
| Yes | 0.880 | 0.017 | 0.749 | 0.001 | 0.848 | 0.077 |
| Age pension for employees (No) |  | . |  |  |  |  |
| Yes | 1.167 | <0.001 | 1.239 | <0.001 | 1.446 | <0.001 |
| Residency (Urban) |  |  |  |  |  |  |
| Rural | 1.060 | 0.072 | 1.235 | <0.001 | 1.383 | <0.001 |
| **Need factors** |  |  |  |  |  |  |
| Household with elderly (No) |  |  |  |  |  |  |
| Yes | 1.363 | <0.001 | 1.275 | <0.001 | 1.241 | <0.001 |
| Household with children (No) |  |  |  |  |  |  |
| Yes | 1.170 | <0.001 | 1.223 | <0.001 | 1.220 | <0.001 |
| Number of members in poor self-rated health (0) |  |  |  |  |  |  |
| 1 | 2.507 | <0.001 | 2.602 | <0.001 | 2.405 | <0.001 |
| >1 | 3.684 | <0.001 | 4.065 | <0.001 | 3.870 | <0.001 |
| **Provincial level variables** |  |  |  |  |  |  |
| Health expenditure per capita | 1.466 | <0.001 | 1.390 | 0.074 | 1.639 | 0.001 |
| Percentage of total government expenditure on health | 1.006 | 0.161 | 1.001 | 0.908 | 1.000 | 0.910 |
| Number of hospital beds per 1 000 population | 0.975 | 0.603 | 0.935 | 0.300 | 0.911 | 0.115 |
| Annal hospitalization rate | 0.978 | 0.131 | 0.993 | 0.442 | 0.990 | 0.378 |
| Average number of medical visits per resident | 0.967 | 0.075 | 1.040 | 0.053 | 0.871 | 0.001 |

Note: AOR-adjusted odds ratio; UEBMI-urban employee basic medical insurance; URRBMI-urban and rural resident basic medical insurance

# Table S11: Factors associated with the incidence of catastrophic health expenditure (CHE) and impoverishing health expenditure (IHE) in eastern developed, central developing, and western underdeveloped regions

| Variables (Reference) | Eastern Region | | Central Region | | West Region | |
| --- | --- | --- | --- | --- | --- | --- |
|  | AOR | p | AOR | p | AOR | p |
| **Year (2013)** |  |  |  | . |  | . |
| 2015 | 1.505 | <0.001 | 1.395 | 0.002 | 1.205 | 0.112 |
| 2017 | 0.714 | 0.005 | 0.536 | <0.001 | 0.548 | <0.001 |
| 2019 | 0.462 | 0.004 | 0.707 | 0.237 | 0.469 | 0.014 |
| **Predisposing factors (household head)** |  |  |  |  |  |  |
| Gender (Female) |  | . |  | . |  | . |
| Male | 0.984 | 0.785 | 0.957 | 0.548 | 0.849 | 0.027 |
| Age (<45 years) |  | . |  | . |  | . |
| 45-54 | 1.227 | 0.027 | 1.329 | 0.005 | 1.119 | 0.231 |
| 55-65 | 1.219 | 0.040 | 1.344 | 0.005 | 1.116 | 0.287 |
| >65 | 1.685 | <0.001 | 1.622 | <0.001 | 1.545 | <0.001 |
| **Predisposing factors (household)** |  |  |  |  |  |  |
| Household size (<3) |  |  |  | . |  |  |
| 3 | 0.536 | <0.001 | 0.52 | <0.001 | 0.583 | <0.001 |
| 4 | 0.418 | <0.001 | 0.325 | <0.001 | 0.499 | <0.001 |
| >4 | 0.338 | <0.001 | 0.345 | <0.001 | 0.386 | <0.001 |
| **Enabling factors (household head)** |  |  |  |  |  |  |
| Educational attainment (Up to primary school) |  |  |  | . |  |  |
| Junior middle school | 0.917 | 0.147 | 0.946 | 0.403 | 0.856 | 0.029 |
| Senior middle school | 0.890 | 0.125 | 0.786 | 0.009 | 0.822 | 0.059 |
| Tertiary education | 0.658 | <0.001 | 0.640 | 0.007 | 0.541 | 0.001 |
| Employment (Unemployment) |  |  |  | . |  |  |
| Employed | 0.571 | <0.001 | 0.589 | <0.001 | 0.656 | <0.001 |
| **Enabling factors (household)** |  |  |  |  |  |  |
| Annual household income per capita (Lowest quintile) |  |  |  | . |  |  |
| Lower | 0.254 | <0.001 | 0.217 | <0.001 | 0.219 | <0.001 |
| Higher | 0.09 | <0.001 | 0.073 | <0.001 | 0.067 | <0.001 |
| Highest quintile | 0.049 | <0.001 | 0.056 | <0.001 | 0.049 | <0.001 |
| Basic medical insurance (Full coverage of UEBMI) |  |  |  | . |  |  |
| None | 0.679 | 0.005 | 0.916 | 0.651 | 1.533 | 0.069 |
| Partial coverage | 1.015 | 0.892 | 1.061 | 0.709 | 1.333 | 0.160 |
| Full coverage of URRBMI | 1.023 | 0.824 | 1.151 | 0.359 | 1.697 | 0.008 |
| Full coverage of mixed | 1.039 | 0.729 | 0.915 | 0.600 | 1.146 | 0.519 |
| Commercial medical insurance (No) |  |  |  |  |  |  |
| Yes | 1.214 | 0.142 | 0.766 | 0.180 | 0.619 | 0.049 |
| Age pension for employees (No) |  |  |  |  |  | . |
| Yes | 0.709 | <0.001 | 0.737 | 0.004 | 1.384 | 0.008 |
| Residency (Urban) |  | . |  |  |  | . |
| Rural | 1.047 | 0.434 | 1.070 | 0.311 | 1.305 | <0.001 |
| **Need factors** |  |  |  |  |  |  |
| Household with elderly (No) |  |  |  |  |  |  |
| Yes | 1.409 | <0.001 | 1.542 | <0.001 | 1.058 | 0.514 |
| Household with children (No) |  | . |  | . |  |  |
| Yes | 0.959 | 0.674 | 0.925 | 0.477 | 1.171 | 0.119 |
| Number of members in poor self-rated health (0) |  | . |  |  |  | . |
| 1 | 3.314 | <0.001 | 3.221 | <0.001 | 2.829 | <0.001 |
| >1 | 4.768 | <0.001 | 4.483 | <0.001 | 5.127 | <0.001 |
| **Provincial level variables** |  |  |  |  |  |  |
| Health expenditure per capita | 1.240 | 0.341 | 1.209 | 0.550 | 1.562 | 0.089 |
| Percentage of total government expenditure on health | 1.008 | 0.417 | 1.010 | 0.380 | 1.015 | 0.055 |
| Number of hospital beds per 1 000 population | 1.058 | 0.570 | 1.191 | 0.122 | 1.102 | 0.372 |
| Annal hospitalization rate | 0.980 | 0.490 | 0.968 | 0.047 | 0.958 | 0.034 |
| Average number of medical visits per resident | 0.985 | 0.704 | 1.003 | 0.941 | 0.930 | 0.340 |

Note: AOR-adjusted odds ratio; UEBMI-urban employee basic medical insurance; URRBMI-urban and rural resident basic medical insurance


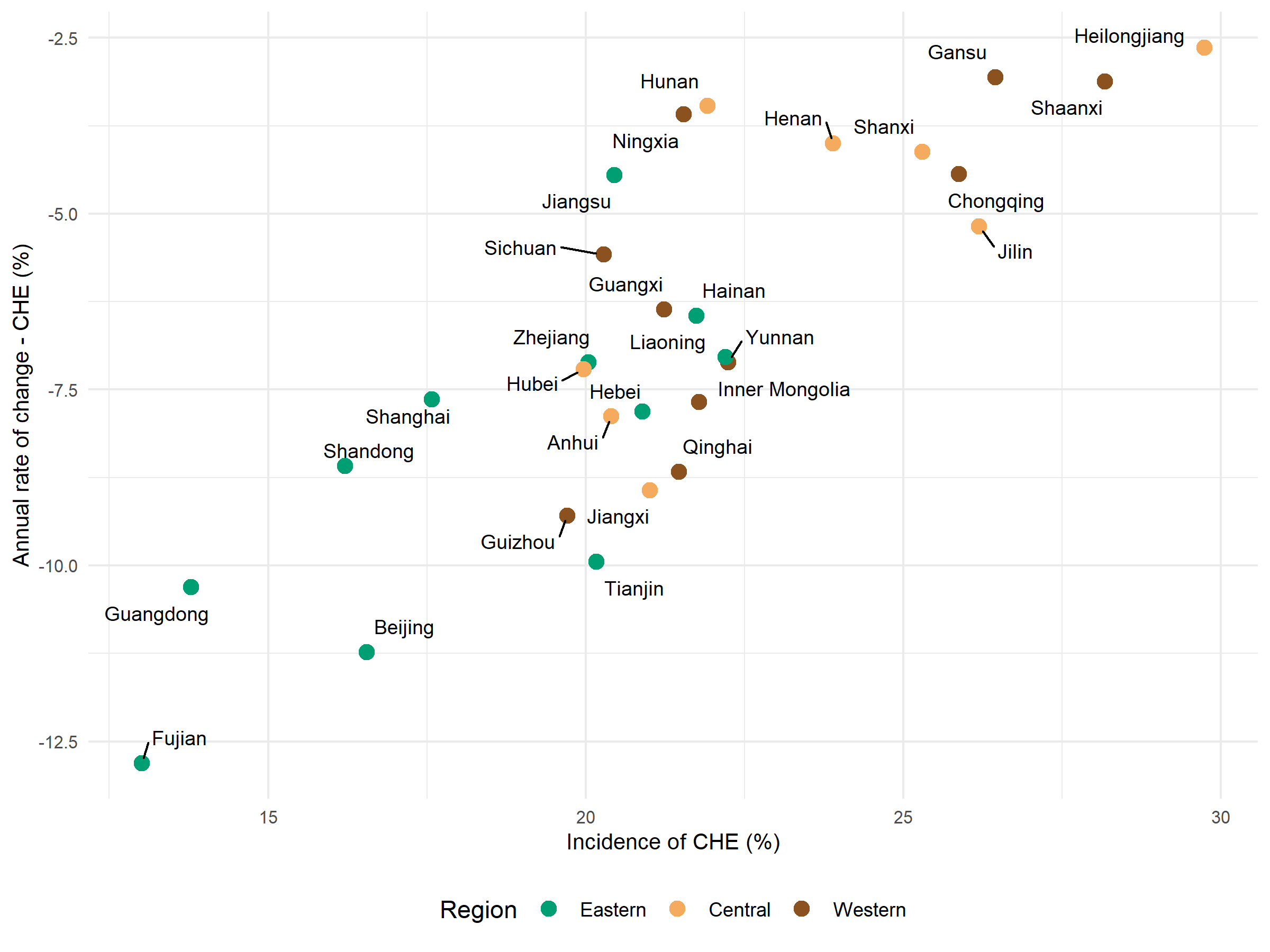


# Figure S1. Incidence in 2019 VS annual rate of change of catastrophic health expenditure (CHE) by province


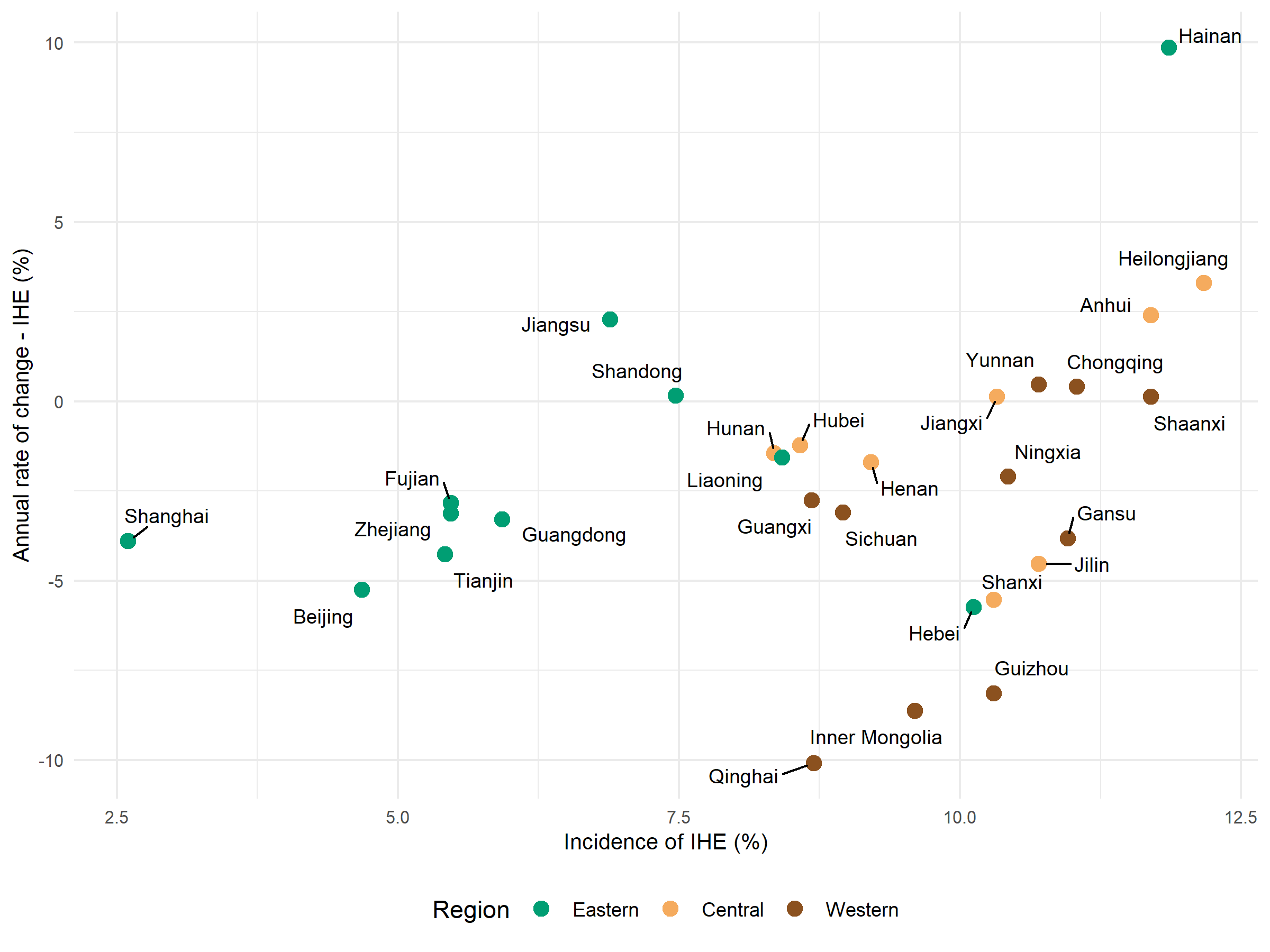


# Figure S2. Incidence in 2019 VS annual rate of change of impoverished health expenditure (IHE) by province

# Annex 1. List of governmental policy documents related to health poverty alleviation

| Policy Title | Date | Issuance Number | URL |
| --- | --- | --- | --- |
| The Decision to Win the Battle against Poverty | 29-11-2025 | Guofa〔2015〕No.35 | https://www.gov.cn/gongbao/content/2015/content_2978250.htm |
| Guidance on Integration of Urban and Rural Residents’ Basic Medical Insurance Systems | 12-01-2016 | Guofa〔2016〕No.3 | https://www.gov.cn/zhengce/content/2016-01/12/content_10582.htm |
| Announcement of National Drug Price Negotiation Results | 20-05-2016 | Guoweibanzhengyaohan〔2016〕No.515 | http://www.nhc.gov.cn/yaozs/s7655/201605/58c5bc1ed0f14c75b8f15f1c149b35f4.shtml |
| Guidance on the Implementation of the Health Poverty Alleviation Project | 21-06-2016 | Guoweicaiwufa〔2016〕No.26 | https://www.gov.cn/xinwen/2016-06/21/content_5084195.htm |
| The 13th Five-Year Plan for Poverty Eradication | 23-11-2016 | Guofa〔2016〕No.64 | https://www.gov.cn/zhengce/content/2016-12/02/content_5142197.htm |
| Implementation of Direct Settlement for Cross-Province Hospitalization Expenses under Basic Medical Insurance | 08-12-2016 | Shenshebufa〔2016〕No.120 | https://www.mohrss.gov.cn/SYrlzyhshbzb/shehuibaozhang/zcwj/201612/t20161215_262040.html |
| National Drug Catalogue for Basic Medical Insurance, Work-Related Injury Insurance, and Maternity Insurance | 23-02-2017 | Renshebufa〔2017〕No.15 | https://www.gov.cn/xinwen/2017-02/23/content_5170392.htm |
| Announcement on the issuance of the "Three Batches" Action Plan for the Health Poverty Alleviation Project | 20-04-2017 | Guoweicaiwufa〔2017〕No.19 | http://www.nhc.gov.cn/caiwusi/s3577c/201704/4eed42903abd44f99380969824a07923.shtml |
| Notice on Implementing Comprehensive Reforms in Public Hospitals | 24-04-2017 | Guoweitigaifa〔2017〕No.22 | http://www.nhc.gov.cn/tigs/s3581/201704/0563e06eff4441ffa9772dc30b487848.shtml |
| Guidance on Advancing the Development of Medical Alliance | 26-04-2017 | Guobanfa〔2017〕No.32 | https://www.gov.cn/zhengce/content/2017-04/26/content_5189071.htm |
| Guidance on Standardizing the Management of Family Doctor Contract Services | 29-09-2018 | Guoweijicengfa〔2018〕No.35 | https://www.gov.cn/zhengce/zhengceku/2018-12/31/content_5435461.htm |
| Three-year Action Plan of Health Insurance for Poverty Alleviation | 30-09-2018 | Yibaofa〔2018〕No.18 | https://www.gov.cn/zhengce/zhengceku/2018-12/31/content_5438703.htm |
| Three-year Action Plan of Health Poverty Alleviation | 19-10-2018 | Yibaofa〔2018〕No.18 | https://www.gov.cn/xinwen/2018-10/19/content_5332738.htm |
| Pilot Program for National Centralized Drug Procurement and Use | 07-01-2019 | Guobanfa〔2019〕No.2 | https://www.gov.cn/zhengce/content/2019-01/17/content_5358604.htm |
| Guidance on Completing the Task of Health Insurance for Poverty Alleviation | 29-09-2019 | Yinaofa〔2019〕No.57 | https://www.gov.cn/zhengce/zhengceku/2019-10/17/content_5456419.htm |
| Plan to Address Key Issues in Ensuring Basic Healthcare Security for Impoverished Populations | 10-07-2019 | Guoweifupinfa〔2019〕No.45 | https://www.gov.cn/zhengce/zhengceku/2019-11/18/content_5453152.htm |
